# Supplementary material for: Trade shocks and trade diversion due to epidemic diseases: Evidence from 110 countries
Source: PLoS One. 2024 May 31;19(5):e0301828. doi: 10.1371/journal.pone.0301828 (PMC11142499; doi:10.1371/journal.pone.0301828)
Supplement: Attachment — Submitted filename: Trade Shocks and Long-term Trade Diversion.doc [file pone.0301828.s002.doc]

Article

**Trade Shocks and Trade Diversion of Epidemic Diseases: Evidence from 110 Countries**

**Naixi Liu ^1^ ,Yu Li ^2^, Mingzhe Jiang ^1^ , Chuanhong Song^5^* and Bangfan Liu^3,4^***

^1^	School of International Economics, China Foreign Affairs University, Beijing, 100024; liunaixi@cfau.edu.cn, jiangmingzhe2016@163.com

^2^ College of Economics and Management, China Agricultural University, Beijing, 100083; 17801065672@cau.edu.cn

^3^ School of public administration Yanshan University, Qinhuangdao 066004 ,China

^4^ Hebei public policy evaluation and Research Center, Qinhuangdao 066004 ,China

^5^ The Center For Economic Research,Shandong University, Jinan 250100, China

*****	Correspondence: mwgtgzy@126.com (C.S.); liubangfan@ysu.edu.cn(B.L)

**Abstract:** The COVID-19 pandemic is already giving a side boost to global trade. Revitalizing global trade after the pandemic is over will be a major theme. Governments have begun to take the problem seriously. China is no exception. In the Party's 20th report, it was clearly proposed to lead the recovery growth of global trade in the post-COVID-19 era. Based on panel data in 1996—2018 of 110 WHO members, use a fixed effect regression model to empirically examine whether epidemic diseases will cause trade shocks and long-term trade diversion, and the results show that: firstly, epidemic disease shocks can lead to negative impacts on a country's trade growth and trade ratio to the world; secondly, with the duration of epidemic diseases growing, the probability of long-term trade diversion effect is also increasing. Further structural analysis based on country heterogeneity proves that the base model has favourable robustness. This provide quite a considerable concern as the shock of COVID-19 lasting, even the experience of China’s achieving positive economic and trade growth under COVID-19 has provided other countries with a way to trade recovery during last three years. However, China also needs to be very alert of the adverse effects due to the outflow of middle and low level industries as well as the long-term trade diversion brought by uncertain epidemics, and consider taking forward-looking supporting policies in advance, it would be too late when the trade damage shows.

**Keywords:** Epidemic Disease; Trade Shocks; Trade Diversion Effect; Resumption of trade

1. Introduction

Since the outbreak of COVID-19, the global social economy has been affected to some extent, and whether this impact is caused by the COVID-19 pandemic or because of other reasons. The answer, is yes. However, to what extent will the COVID-19 pandemic affect the future of global society and economy, especially trade, or even lead to trade backsliding? These questions cannot be answered immediately, but must be answered in depth. Our Party and government also attach greater importance to these problems. In the Party's 20th report, it was clearly proposed to lead the recovery growth of global trade in the post-COVID-19 era.

The Fifth Plenary Session of the 19th Central Committee of the CPC made it clear that we will improve the effectiveness of national governance, improve the systems and mechanisms for preventing and defusing major risks, and significantly enhance our capacity to respond to public emergencies. As a public health emergency, epidemic disease is a kind of natural disaster that is difficult to predict in advance and irresistible. It often brings strong external impact on economic development, especially on international economic activities such as import and export trade and cross-border investment, and even impacts the stability of a country's industrial structure by causing long-term trade diversion effect. The long-term economic growth of the affected countries will be severely affected.

The outbreak of the COVID-19 pandemic in December 2019 has brought economic activities around the world to a standstill, and foreign trade has been Mired in difficulties. Under the condition of epidemic disease impact and unstable external economic environment, Chinese foreign trade deficit gradually expanded, and then it was alleviated after one quarter. With the strengthening of China's macro policies, the domestic epidemic has been effectively controlled, China's foreign trade has been stable and improved, and its economy has gradually recovered. China is the only country among the world's major economies to achieve positive growth in 2020.In 2021, the national economy will continue to maintain a steady recovery, production demand will continue to recover, and economic growth is expected to achieve the growth target of more than 6%.

However, there are still significant uncertainties in the current situation of the global epidemic. In 2020, other countries in the world were generally ineffective in responding to the epidemic. The measures taken by countries to stabilize trade have yet to yield significant results. World trade has contracted significantly and national economies have suffered a huge impact. The world is still in the stage of regular epidemic prevention and control, and the sudden change of the COVID-19 has made the epidemic prevention and control even worse. Due to the large population base, China's vaccination rate is much lower than that of countries such as the UK and the US, and there is still a big gap between 70 and 80 percent of the targeted vaccination rate. China still faces the risk of imported epidemics and repeated epidemics in small areas in China. This also requires us, on the basis of summarizing historical experience and facts, to search for more solid general law conclusions through empirical research, so as to make certain judgments and predictions on the impact that the normalization of COVID-19 may bring to the foreign trade development of all countries in the world (especially other countries except China) and whether there will be long-term trade transfer in the future. In general, there are two limitations in previous relevant studies, which make them unable to effectively answer these two important questions.

First, there are few empirical studies on the impact of epidemics on import and export trade. Some scholars have studied the impact of epidemics on trade, mostly focusing on the temporary stagnation of trade in the short term (Huang Yueheng et al., 2004;Leadon and Herholz, 2009;Shi Changhua and Gong Hanying, 2013), but only a few scholars have qualitative discussion on whether such shocks will form short-term trade transfer effect (Huang Zhaoyin, 2003;Shao Bai et al., 2019).

Second, there are few empirical studies on the long-term trade diversion effect caused by epidemics. Most scholars have discussed trade stagnation and short-term trade diversion in the impact of specific epidemics from a qualitative perspective (Huang Zhaoyin, 2003;Leadon and Herholz, 2009;Shi Changhua and Gong Hanying, 2013;Perrings et al., 2018;Shao Bai et al., 2019), and few scholars have conducted empirical studies on this issue, especially on the long-term trade transfer effect, which cannot provide relatively general argumentative support for the above issues.

In order to make up for the above limitations and seek a relatively general research demonstration, this paper innovatively uses the balanced panel data of disease records and macro data of 110 member countries released by the World Health Organization from 1996 to 2018 to make an empirical test on whether the disease impacts a country's trade and whether it has a long-term trade transfer effect. The impact of epidemic and non-epidemic diseases on different countries is also discussed. The first part of this paper is the introduction; The second part is literature review; The third part is the theoretical framework, in which we elaborate the impact mechanism and path of the disease that may impact trade and cause the long-term trade diversion effect. The fourth part is model construction, data selection and variable interpretation. The fifth part is the estimation results and discussion; The sixth part is to further group countries to carry out structural comparative analysis and robustness test. The seventh part is the conclusion and policy recommendations.

2. Literature review

2.1 Research on the impact of epidemics on trade and the effect of trade diversion

The World Health Organization's "Building a Secure Future: Global Public Health Security in the 21st Century" report notes that disease outbreaks can cause significant economic losses by disrupting trade and travel. Some scholars also studied the impact of epidemics on trade.

Huang Zhaoyin (2003) analyzed the impact of SARS on China's trade and found that the trade fluctuation was small in the month of SARS outbreak. However, due to the lag, business activities were postponed or even cancelled in the following months, the export threshold of Chinese products was raised, the export share decreased, and foreign trade was difficult to carry out. Huang Yueheng et al. (2004), based on the perspective of importing countries, linked the setting of technical trade barriers for different countries with the probability of epidemic occurrence in exporting countries, and raised the technical import threshold for countries with high probability of epidemic occurrence to reduce the risk of epidemic impact on domestic trade.Leadon and Herholz (2009) illustrated the historical evidence of the introduction of pathogens into disease-free areas by the horse product trade, and strongly demonstrated that the disease caused the short-term stagnation of the horse trade. Shi Changhua and Gong Hanying (2013) reviewed various international public health emergencies, and found that the health control measures taken by various countries for personnel, entry-exit goods and vehicles brought huge losses to various economies, especially foreign trade. Perrings et al. (2018) believed that the emergence and spread of infectious diseases were closely related to the growth of trade network. When the epidemic occurred, the epidemic could quickly spread to all directions of the trade network through trade activities, making importers become the driving force in determining the trade flow, and the import activities avoided the affected countries. Shao Bai et al. (2019) analyzed the impact of international public health emergencies on countries' trade: the interruption of foreign economic exchanges and the reduction or delayed transfer of foreign trade orders in the affected countries; Neighboring countries of the affected countries have seen a significant shift in trade, resulting in a substantial increase in export demand.

2.2 Other relevant studies on impact on trade and formation of trade transfer effect

In general, in addition to the epidemic factor, there are many discussions on the impact of other factors on trade and the formation of trade transfer effect in previous literature. There are mainly three reasons.

*Trade agreements and changes in trade barriers*

Clausing (2001) analyzed the influence of the FTA on the trade patterns of the two countries, and the results showed that the FTA had a trade creation effect, but no trade transfer effect. Bohara et al. (2004) verified the view that "trade integration may exacerbate inefficiency" and found that trade was indeed transferred to free trade area partner countries, but this trade transfer effect was internalized and weakened due to the disappearance of trade barriers. Guo Yan and Li Xiubin (2004) studied the zero-quota and zero-tariff textile trade agreements signed by the United States with some developing countries on the basis of the North American Free Trade Agreement (NAFTA), and observed that the traditional textile exporting countries in Asia suffered losses from trade transfer. Zhang Bin and Zhang Shu (2005) further analyzed the trade transfer of various commodities in the United States under the influence of NAFTA from 1994 to 2003, and found that there was an annual discontinuous trade transfer effect on labor-intensive manufactured goods. Magee (2008) estimated the impact of regional trade agreements (Rtas) on trade flows with the econometric method, and the results showed that Rtas had a significant predictive signal effect and would continue to affect trade in the next 11 years. Shen Hao and Yang Yong (2008) analyzed the trade transfer effect of African economic integration and found that the intra-community trade scale was too small to change the trade flow during 1999-2005.Datta and Kouliavtsev (2009) studied the effects of wages, tariffs and exchange rates on the import structure of American textiles before and after the establishment of NAFTA, and the results showed that the trade creation effect was significant, while the trade diversion effect was not. Sun and Reed (2010) conducted OLS estimation on the trade gravity model and calculated that there was a trade transfer effect among the members of the free trade Agreement, and agricultural trade increased substantially. Baylis and Perloff (2010) found that when voluntary price constraints were strong, Mexico shifted its tomato export from the United States to Canada, while Canada increased its tomato product export to the United States. Cao Liang et al. (2013) calculated the trade flow of China-Asean Free trade area by combining the trade gravity model, and demonstrated that there is no import trade transfer in China's bilateral relationship, but only trade creation. Yang and Martinez (2014) also found that the trade agreement has brought huge trade creation to ASEAN. Dai et al. (2014) collated historical data and came to the conclusion that free trade agreements transfer trade from non-member countries to member countries. Cheong et al. (2015) explored the impact of preferential trade agreements on trade and observed that preferential trade agreements could weaken trade creation and shield the trade transfer effect of future trade agreements, thus minimizing inter-regional trade losses. Magee (2016) estimated the general equilibrium effect of the European Community and found that the trade creation generated by the customs union was more than twice the trade transfer. Xu Fen and Liu Hongman (2017) calculated that there was no trade transfer effect in China's agricultural exports to different free trade zones based on the trade gravity model and import demand model.

*International trade disputes*

Brenton (2001) summarized the anti-dumping actions of various countries against the EU, and observed that the anti-dumping policy would lead to the transfer of the importing trade of the suing country to non-EU suppliers. Chen Hanlin (2008) found that anti-dumping actions initiated by the United States against China greatly reduced China's exports to the United States, and part of the reduction may be transferred to the European Union. Xiang Hongjin (2008) found that the export of similar products from South Korea, India and other countries to the United States increased in the same period. Liu Gravi and Shao Min (2009) focused on Indian antidumping, listed the differences of Chinese product exports to India, and concluded that the transfer effect of Chinese product trade with comparative advantage was small. Park (2009) focused on China's anti-dumping investigation, built a panel model and calculated that China's anti-dumping protection has significant trade inhibition effect and trade diversion effect. Sun Ming and Yang Shihui (2011) took international countervailing cases from 1993 to 2007 as the research object and estimated that countervailing complaints caused the transfer of product imports from the accused country to the non-accused country. Liu Graviti and Cao Jie (2011) started from the product categories of EU anti-dumping against China and found that the trade diversion effect was inconsistent with the intensity of anti-dumping, and then analyzed that the differences came from product competitiveness and product dependence. Zhang Yong (2013) made use of the anti-dumping cases initiated by the United States from 1994 to 2008 to demonstrate that the complaint of the United States can only increase the import market share of the non-respondent country, without the trade transfer effect.Hao Liang (2017) established an anti-dumping duty model and analyzed the optimal anti-dumping duties made by manufacturers at the same time or successively from the perspective of microeconomics, and calculated that there was no significant correlation between the level of the tax and the strength of the trade transfer effect.

*Economic crisis impact*

Headey (2011) sorted out and observed the changes of international grain export volume and export price before the outbreak of the food crisis in 2008, and found that the surge of grain price lagged behind the rise of export volume, and the surge of food demand in the international market caused 15 to 27 food price changes worldwide, which then led to the change of a country's food import demand. Liu Hongguang et al. (2019) sorted out the industrial trade transfer structure of major countries and regions before and after the 2008 financial crisis and argued that China was the largest net industrial transfer country, while the EU, the United States and other places gradually became the destinations of service trade transfer.

(3) Summary of literature

In conclusion, the conclusion of trade agreements (or changes in trade barriers), international trade disputes and economic crises can affect trade, while the conclusion of trade agreements and economic crises will lead to medium - and long-term trade diversion. However, in previous studies, there are relatively few empirical studies on whether epidemics can impact trade and cause long-term trade transfer effect. Only some scholars have conducted qualitative analysis on trade stagnation and short-term trade transfer caused by specific epidemics, and no general conclusions can be drawn on this issue. This paper tries to provide a supplementary answer to this question.

3. Theoretical framework

We classify epidemics into epidemic diseases and non-epidemic diseases, deconstruct and analyze the impact mechanism of epidemics on trade and long-term trade transfer, establish a theoretical framework, and put forward two empirical hypotheses based on this. Since the mechanism of the impact of non-epidemic diseases and epidemic diseases on trade is the same, and the impact of epidemic diseases on trade is smaller than that of epidemic diseases, we take epidemic diseases as an example to discuss the theoretical mechanism of the impact on trade and the long-term trade diversion.

First of all, for inter-industry and inter-multinational trade, there are three impacts (as shown in Figure 1) :

Path 1: During the outbreak of epidemic disease, enterprises will spontaneously restrict production or stop work due to the panic and collective action to prevent and control the spread of epidemic disease. Foreign suppliers or downstream manufacturers will be forced to cooperate with enterprises in other countries. The trade chain of affected countries will be interrupted, impacting foreign trade and forming short-term trade diversion effect. However, with the end of the epidemic, enterprises in the affected countries tend to reduce their costs due to overcapacity, and the country's products and services will recover a certain degree of international competitiveness, resulting in new trade creation and transfer effects in the international market, thus slowing down the original short-term trade transfer. However, affected by the shrinking demand during the epidemic, when production resumption causes overcapacity, it is difficult to realize such production resumption, and enterprises cannot recover their original international market share, resulting in long-term trade transfer effect.

Path 2: The outbreak of epidemic disease has caused "health concerns" in the international market for products and services exported by the affected countries. For example, import restrictions or higher import standards have been imposed on products and services exported by the affected countries, which has impacted the export trade of the affected countries (for example, in the case of the outbreak of COVID-19 in 2020, South Korea, Indonesia and other countries have taken measures to restrict the import of agricultural products from China), the original trading partners have turned to trade with "safe countries", and the affected countries have been forced to form a long-term trade diversion effect.

Path (3) : With the increase of the duration of the epidemic and the expansion of the spread countries, it is easy to form a rotation to prolong the time delay of the epidemic spread. At this time, the international market demand is at a long-term low, the impact on the trade will be expanded, and the probability of the short-term trade substitution to evolve into the long-term trade transfer effect will also be increasing.

**Figure 1.** Logical diagram of the impact of the disease on trade and the formation of long-term trade diversion effect.

Note: The solid arrows indicate the transmission mechanism of the impact of epidemic diseases on trade and the long-term trade diversion effect;The dotted line indicates that there is a correspondence between the onset and the duration of the epidemic.

Secondly, for intra-industry and intra-multinational trade, when the whole industry is not in recession, it is more difficult to replace intra-industry and intra-multinational trade. Although when the epidemic occurs, short-term trade replacement will be sought and short-term trade transfer effect will be generated, and the possibility of long-term trade transfer effect will be relatively small after the epidemic ends. However, when the whole industry falls into recession, even if it cannot find a long-term trade replacement during the epidemic period, the trade of the industry will remain at a low level for a long time during the recession, which will still impact the distribution structure of the original trade chain and eventually form a long-term trade transfer effect.

Therefore, based on the above theoretical mechanism analysis, we propose two hypotheses to be tested:

Empirical hypothesis 1: Epidemic diseases will have an impact on a country's trade.

Empirical hypothesis 2: The impact of epidemic diseases have a long-term trade diversion effect, and the duration of epidemics has a nonlinear relationship with the long-term trade diversion effect.

4. Model construction, data selection and variable interpretation

4.1 Model construction

kAccording to the two empirical hypotheses proposed above and referring to previous literatures, we set the model as follows:

Equation for estimating trade growth impact Ⅰ, Ⅱ :

$\begin{matrix} ExportGrowth_{it}=\frac{Export_{it}}{Export_{i,t-1}}=\beta_{0}+\beta_{1}EDI_{it}+\beta_{2}NEDI_{it}+\beta_{3}EDITime_{it}+\beta_{4}EDITime_{it}^{2} \\ +\beta_{5}NEDITime_{it}+\beta_{6}NEDITime_{it}^{2}+\beta_{7}RTAIn_{it}+\beta_{8}Disputes_{it}+\sum_{k=9}^{m}\beta_{k}Z_{it}+\eta_{it} \end{matrix}$(Ⅰ)

$\begin{matrix} TradeGrowth_{it}=\frac{Trade_{it}}{Trade_{i,t-1}}=\beta_{0}+\beta_{1}EDI_{it}+\beta_{2}NEDI_{it}+\beta_{3}EDITime_{it}+\beta_{4}EDITime_{it}^{2} \\ +\beta_{5}NEDITime_{it}+\beta_{6}NEDITime_{it}^{2}+\beta_{7}RTAIn_{it}+\beta_{8}Disputes_{it}+\sum_{k=9}^{m}\beta_{k}Z_{it}+\gamma_{it} \end{matrix}$ (Ⅱ)

Trade ratio impact estimation equation Ⅲ and Ⅳ :

$\begin{matrix} ExportRatio_{it}=\frac{Export_{it}}{Export_{wt}}=\beta_{0}+\beta_{1}EDI_{it}+\beta_{2}NEDI_{it}+\beta_{3}EDITime_{it}+\beta_{4}EDITime_{it}^{2} \\ +\beta_{5}NEDITime_{it}+\beta_{6}NEDITime_{it}^{2}+\beta_{7}RTAIn_{it}+\sum_{k=8}^{m}\beta_{k}Z_{it}+\nu_{it} \end{matrix}$ (iii)

$\begin{matrix} TradeRatio_{it}=\frac{Trade_{it}}{Trade_{wt}}=\beta_{0}+\beta_{1}EDI_{it}+\beta_{2}NEDI_{it}+\beta_{3}EDITime_{it}+\beta_{4}EDITime_{it}^{2} \\ +\beta_{5}NEDITime_{it}+\beta_{6}NEDITime_{it}^{2}+\beta_{7}RTAIn_{it}+\sum_{k=8}^{m}\beta_{k}Z_{it}+\tau_{it} \end{matrix}$ (Ⅳ)

Where, i represents the country, t represents the corresponding year, and w represents the world total level.

The construction of the above model is mainly based on two considerations:

First, according to empirical hypothesis 1, we use the trade growth rate of a country in a certain year as the dependent variable of models I and II to estimate the impact of epidemic disease on a country's trade. This is because trade growth tends to have a certain inertia, and the impact of epidemics on a country's foreign trade is generally reflected in the impact on its original growth trend rather than the absolute value.

Secondly, according to empirical hypothesis 2, we use the ratio of a country's trade volume to the world's total trade volume in a certain year as the dependent variable of models Ⅲ and Ⅳ to estimate the trade transfer effect. This is because in the short term, it is difficult for the world trade structure to undergo sudden changes. It is assumed that in the case of equilibrium without trade transfer effect, the trade volume of a country should keep growing in the same proportion as the total world trade volume. The inconsistency between the growth of a country's trade volume and the world's total trade volume may be due to the fact that the country's original trade was replaced by the trade of other countries, or the country failed to enter the new trade market. It is reasonable to believe that the change of the trade ratio of a country's total trade volume to the world's total trade volume can reflect the change of the country's trade structure. That is, there are broad trade diversion effects.

4.2 Data selection

In this paper, panel data from WHO member countries are used as the sample for the metrological tests. Disease related data were collated according to the news on diseases released by the World Health Organization from 1996 to 2018. A total of 59 diseases were involved, which were clearly defined as epidemic diseases (21 epidemic diseases were involved in this paper). We consider non-epidemic diseases in the list of diseases that are not defined as epidemic diseases (38 non-epidemic diseases are covered in this article).The trade and macro data of the corresponding countries are respectively from the WTO database and the World Bank database. After excluding the countries with serious data shortage, the balance panel data of 110 countries were obtained as the econometric test samples.

4.3 Variable interpretation

*Explained variables*

ExportGrowth_it_ And ExportRatio_it_ Represents, respectively, the growth rate of exports and the ratio of exports to the world's total exportsit(The value of a country's trade, unit: USD million) and Export_wt_(World exports, unit: million US dollars).

TradeGrowth_it_ And TradeRatio_it_ Respectively represents the growth rate of imports and exports and the ratio of imports and exports to the world's total imports and exports, by Trade_it_(A country's Trade imports and exports, unit: million US dollars) and trade_wt_(World import and export, unit: millions of US dollars).

*Variable of explanation*

EDI_it_: Epidemic Disease Impact represents the impact of epidemic diseases in country i in Year t. This variable is calculated using the weighted sum of the case fatality rate of different epidemic diseases and the square value of the affected area (number of affected countries) of the current year on the basis of 0-1 dummy variables. We used the case fatality rate to reflect the economic impact of epidemic diseases, because when there is a probability of death, the social panic of epidemic diseases will be intensified, which will lead to spontaneous stagnation of economic activities, and make traders in affected countries voluntarily reduce their trade activities. The squared weighting is used because when multiple countries are hit by a pandemic, international trade tends to spiral downward and become more skewed towards only "safe countries", exacerbating trade stagnation. When there is no epidemic disease, the value is 0.

NEDI_it_: Non-Epidemic Disease Impact represents the impact of non-epidemic diseases in country i in year t. This variable is calculated from the weighted sum of different areas of influence (number of affected countries) of non-epidemic diseases on the basis of 0-1 dummy variables. We weighted only the number of affected countries because nonepidemic diseases are less contagious and less likely to cause widespread social panic. When there is no non-epidemic disease, the value is 0.

EDITime_it_: Represents the duration of epidemic disease (unit: year).The value of variable in the year of epidemic outbreak is 1, and the value of corresponding variable is increased by 1 for each additional year of duration thereafter .Considering the possible non-linear relationship between the duration of the outbreak and the effects of trade diversion, both the first and quadratic forms of EDITime_it_ are added to the estimation equation.

NEDITime_it_: Represents the duration of non-epidemic disease (unit: year).The value of the variable in the year of the outbreak of non-epidemic disease is 1, and the value of the corresponding variable is increased by 1 for each additional year of the duration thereafter. Like EDITime, both the first and quadratic forms of NEDITime_it_ are added to the estimation equation.

*Variable of control*

RTAIn_it_: Regional Trade Agreements Increase (Regional Trade Agreements Increase) is defined as the increase in the number of regional trade agreements signed by country i in year t. The data is from the WTO Trade agreement database, which is used to control the possible impact of new trade agreements signed by the country on trade. In other words, when the number of agreements signed by a country changes, there may be changes in trade growth and trade transfer effects caused by trade agreements.

Disputes_it_: Defined as the number of cases in which Country i was the litigant in international trade disputes in year t. The number of trade disputes in all the years between the time a dispute was raised and its settlement was reached is added to the value of this variable. Considering that the impact of international trade disputes on a country's trade is mainly manifested as the impact on the change of trade growth rate in the current year (Chen Hanlin, 2008;Xiang Hongjin, 2008;Sun Ming and Yang Shihui, 2011), but there is no inevitable causal logical connection with trade transfer (Zhang Yong, 2013;Hao Liang, 2017). Therefore, we only added international trade dispute control variables into models Ⅰ and Ⅱ.

Z_it_: is a group of control variables added by referring to previous research literatures, mainly to control other macroeconomic variables affecting trade changes, as shown in Table 1 below.

**Table 1.** Control variables -- macroeconomic variables (unit: percentage)R

| variable | meaning | Variable selection Description |
| --- | --- | --- |
| *GDPGrowth* | GDP growth rate | Reflecting national income and national economic development, there is an endogenous relationship between GDP growth and trade growth of a country |
| *POPGrowth* | Population growth rate | Population growth drives demand growth, which in turn boosts import consumption |
| *MGrowth* | Broad money growth rate | Currency increase can reduce short-term nominal interest rate, but also make a country's currency relative depreciation, expand export trade and reduce import trade; and vice versa |
| *R* | Effective interest rate | The rise of real interest rate leads to capital inflow and relative currency appreciation, which expands import trade and reduces export trade, while the rise of savings under liquidity preference will expand trade surplus.And vice versa |
| *Agriculture* | Agricultural value added as a percentage of GDP | Reflecting the structure of the primary industry and influencing the export structure of agricultural products is particularly important for agricultural powers |
| *Industry* | Industrial added value as a percentage of GDP | To reflect the structure of the secondary industry and affect the export structure of industrial products is particularly important for industrial powers |

(4) Endogeneity problems

Epidemics can have an impact on trade and form a long-term trade transfer effect. It is worth considering whether epidemics are endogenous variables that affect a country's trade growth rate and trade ratio. However, as a natural disaster that is difficult to predict in advance, epidemic disease is a dummy variable in this paper. The interruption or stagnation of trade caused by epidemic disease is the direct impact of epidemic disease on trade, and it is not easy to have other effective exogenous variables that can cause epidemic disease and then have an indirect impact on trade. On the other hand, in the case of trade health regulation, the further expansion of the epidemic is often not due to the development of a country's trade, but to the cross-border and cross-regional movement of people in an ineffective way to prevent transmission. Therefore, the possible endogeneity of epidemic variables can be reasonably ignored.

5. Estimation results and discussion

5.1 Descriptive statistics of variables

The descriptive statistics of variables in the estimation equation are shown in Table 2.

**Table 2**. Statistical Table of variable description (Total samples: 2530).

| variable | The mean | Standard deviation | Minimum value | Maximum value |
| --- | --- | --- | --- | --- |
| *ExportGrowth* | 1.0923 | 0.2392 | 0.2941 | 4.7143 |
| *TradeGrowth* | 1.0842 | 0.1664 | 0.3581 | 1.9407 |
| *ExportRatio* | 0.0048 | 0.0144 | 3.50 e-07 | 0.1375 |
| *TradeRatio* | 0.0049 | 0.0156 | 1.85 e-06 | 0.1549 |
| *EDI* | 363.1312 | 1230.729 | 0 | 5854.32 |
| *NEDI* | 0.2217 | 1.3007 | 0 | 18 |
| *EDITime* | 0.4043 | 1.1682 | 0 | 14 |
| *NEDITime* | 0.0806 | 0.3894 | 0 | 7 |
| *RTAIn* | 0.3162 | 1.0346 | 0 | 28 |
| *Disputes* | 0.5711 | 2.1784 | 0 | 28 |
| *GDPGrowth* | 3.9657 | 4.2499 | 30.1451 | 88.9577 |
| *POPGrowth* | 1.5142 | 1.3285 | 9.0806 | 8.1179 |
| *MGrowth* | 17.4821 | 85.2695 | 99.8637 | 4105.573 |
| *R* | 7.3454 | 10.6454 | 93.5135 | 130.345 |
| *Agriculture* | 14.1097 | 12.3015 | 0.0249 | 79.0424 |
| *Industry* | 26.2827 | 11.2967 | 3.1502 | 74.1130 |

5.2 Estimation process and results

Due to significant country heterogeneity and macroeconomic cycle effects, country and year fixed effects should be controlled in economic logic. However, considering the possible partial sampling effect of sample data, we still need to test its measurement characteristics. The fixed effects model and random effects model were used to estimate the regression equation, and then the Hausmann test was used to make the selection judgment. The results show that (as shown in Table 3) it is more appropriate to select the fixed-effect model.

**Table 3**. Hausmann test results.

| model | Ⅰ | Ⅱ | Ⅲ | Ⅳ |
| --- | --- | --- | --- | --- |
| χ^2^value | 91.18^***^ | 124.96^***^ | 143.55^***^ | 145.87^***^ |
| P values | 0.000 | 0.000 | 0.000 | 0.000 |

Note:^*^ *p < 0.05,* ^**^*p < 0.01,* ^***^*p < 0.001;*The values in parentheses are t-values.

The macroeconomic variables used in our estimation all have first-order derivative properties (the discrete form is reflected in the growth rate or ratio), and since the number of estimated cross-section samples is much larger than the number of years, it is no longer necessary to conduct a separate unit root test (Anderson and Cheng, 1982;Bai, 2009).Therefore, we estimate the fixed effects of equations (I) to (IV) respectively, and the estimated results are shown in Table 4, which mainly include the following conclusions:

(1) Epidemic EDI had a significant negative impact on both ExportGrowth and TradeGrowth of a country's export trade, indicating that epidemic EDI could have an impact on a country's trade, and the empirical hypothesis was confirmed. Further comparison of the regression coefficient shows that the impact of epidemic diseases on export trade is weaker than the overall impact on import and export trade, which may be caused by more countries with international trade deficit in the sample of this paper. In the sample period, the average number of countries with international trade deficits was 83 out of 110 WHO member States. This also means that the impact of the pandemic on trade deficit countries may be stronger than that on trade surplus countries.

The non-epidemic epidemic disease NEDI has no significant impact on the ExportGrowth and TradeGrowth of a country's export trade, indicating that the non-epidemic epidemic disease has no significant impact on a country's trade.

(2) EDI of epidemic disease has a significant negative impact on both ExportRatio and TradeRatio of a country's export to the world ratio and total import and export to the world ratio, indicating that epidemic disease impact has a long-term trade diversion effect, which is confirmed by the two empirical hypothesis. By further comparing the regression coefficient, we can see that the impact of epidemic disease on the export to the world ratio is greater than that of the total import and export to the world ratio, indicating that the long-term trade transfer effect of epidemic disease on export trade is stronger.

The influence of NEDI on the ExportRatio of a country's export volume and the exportratio of its total import and export volume in the world is not significant, indicating that the impact of NEDI does not have a long-term trade diversion effect.

(3) By observing the regression coefficient of epidemic duration EDITime, it can be found that the primary type has a positive impact on trade growth and trade ratio, while the secondary type has a negative impact on trade growth and trade ratio, indicating that the negative impact of epidemic duration on trade has a concave feature.This indicates that initially, with the stabilization of the epidemic and the improvement of confidence, especially the rapid recovery of the epidemic in the short term, the impact of the epidemic on trade was alleviated. On the contrary, the demand for epidemic prevention trade during the epidemic and the reported rebound in the recovery period resulted in a small increase in the short-term trade. However, with the further increase of the duration of the epidemic, the confidence to overcome the epidemic has entered a new unstable state, and as the epidemic spreads to more countries, the external environment deteriorates, the short-term trade substitution gradually transitions to the long-term trade transfer, and the impact of the epidemic on trade continues to intensify.

NCDS have a significant negative impact on the trade ratio, which may be due to the fact that NCDS have a small range of infection and cannot affect the external environment, while the short-term decline in their own trade makes the ratio decrease. Because of the relatively short duration of non-epidemic diseases, secondary forms do not show significant effects.

(4) RTAIn has a significant positive effect on TradeGrowth of a country's import and export trade, which indicates that the growth of regional trade agreements brings about trade creation effect. RTAIn also has a significant positive impact on the TradeRatio of a country's total import and export trade in the world, indicating that the trade creation effect brought by the growth of regional trade agreements is stronger than the trade substitution and crowding out effect, which is consistent with the conclusions of most previous studies.

(5) GDP growth rate, monetary growth rate and real interest rate eall have a significant impact on a country's trade growth, but have no significant impact on a country's trade to the world ratio. Among them, GDP growth can promote a country's trade growth, which is fully in line with our theoretical expectations when we select control variables. The rise of money growth will have a negative inhibitory effect on trade growth, because the demand response is faster and more flexible than the supply response. The short-term inhibitory effect of money growth on import trade is greater than the short-term promotion effect on export trade. The rise of real interest rate will also have a negative inhibitory effect on trade growth, indicating that the negative impact on trade through capital flow and exchange rate is greater than the positive impact on trade through investment and savings growth.

(6) The proportion of agricultural added value to GDP has a significant negative impact on a country's trade ratio. From a general point of view, the faster the export of agricultural products (natural resource-intensive products) is growing, the slower the export of industrial products is growing. In the past two decades, the proportion of agricultural trade in international trade has been declining, and the trade of industrial goods and services has taken an increasing share, which makes the proportion of trade of agricultural exporting countries in the world decreased.

(7) The ratio of industrial added value to GDP has a positive and significant impact on a country's trade growth, which indicates that the rise of a country's industrial output value can increase the export of industrial products, thus promoting the trade growth. Especially for those countries dominated by industrial exports (labor-intensive), this positive correlation is more consistent with the general fact.

**Table 4.** Estimation results table

|  | Model I | Model II | Model Ⅲ | Model Ⅳ |
| --- | --- | --- | --- | --- |
|  | *ExportGrowth* | *TradeGrowth* | *ExportRatio* | *TradeRatio* |
| *EDI* | 0.0000125^**^ | 0.0000150^***^ | 0.000000444^***^ | 0.000000428^***^ |
|  | (2.82) | (5.18) | (5.26) | (5.52) |
| *NEDI* | 0.00635 | 0.00570 | 0.000147 | 0.0000820 |
|  | (1.40) | (1.92) | (1.70) | (1.03) |
| *EDITime* | 0.0261^**^ | 0.0270^***^ | 0.00138^***^ | 0.00126^***^ |
|  | (2.68) | (4.22) | (7.41) | (7.37) |
| *EDITime^2^* | 0.00282^*^ | 0.00300^***^ | 0.0000780^***^ | 0.0000730^***^ |
|  | (2.41) | (3.91) | (3.49) | (3.55) |
| *NEDITime* | 0.0173 | 0.0318 | 0.00199^***^ | 0.00173^***^ |
|  | (0.63) | (1.78) | (3.82) | (3.61) |
| *NEDITime^2^* | 0.00721 | 0.0105^**^ | 0.000225 | 0.000169 |
|  | (1.18) | (2.61) | (1.93) | (1.58) |
| *RTAIn* | 0.00738 | 0.00759^*^ | 0.000161 | 0.000178^*^ |
|  | (1.59) | (2.49) | (1.87) | (2.25) |
| *Disputes* | 0.00210 | 0.00374 | - | - |
|  | (0.58) | (1.57) | - | - |
| *GDPGrowth* | 0.0214^***^ | 0.0181^***^ | 0.000000637 | 0.00000305 |
|  | (18.81) | (24.22) | (0.03) | (0.15) |
| *POPGrowth* | 0.00655 | 0.0102^*^ | 0.0000474 | 0.0000401 |
|  | (0.96) | (2.26) | (0.36) | (0.33) |
| *MGrowth* | 0.000103 | 0.0000740^*^ | 0.000000453 | 0.000000280 |
|  | (1.86) | (2.04) | (0.43) | (0.29) |
| *R* | 0.00530^***^ | 0.00436^***^ | 0.000000844 | 0.000000286 |
|  | (9.85) | (12.34) | (0.08) | (0.03) |
| *Agriculture* | 0.000116 | 0.000209 | 0.0000961^***^ | 0.0000958^***^ |
|  | (0.10) | (0.27) | (4.25) | (4.61) |
| *Industry* | 0.00302^*^ | 0.00350^***^ | 0.0000206 | 0.0000254 |
|  | (2.43) | (4.30) | (0.87) | (1.17) |
| Term of constant | 0.973^***^ | 0.971^***^ | 0.00651^***^ | 0.00679^***^ |
|  | (22.91) | (34.85) | (8.06) | (9.14) |
| Number of samples | 2530 | 2530 | 2530 | 2530 |

Note:^*^ *p < 0.05,*^**^ *p < 0.01,*^***^ *p < 0.001;*The values in parentheses are t-values.

6. Structural analysis and robustness test

In order to better understand the impact of the epidemic on different types of countries, we further carried out structural analysis and at the same time carried out some robustness tests.

6.1 Comparison of the impact of epidemics on trade between large and small countries

For big countries, due to their dominant position in international trade, epidemics are more likely to have an impact on their trade. The complex industrial structure of big countries makes it more difficult to recover from the impact, which may lead to long-term trade diversion effect. For small countries, the impact of epidemics on trade growth is relatively small. We used the 2018 median GDP of each country published by the World Bank to classify large and small countries, and estimated the total impact of the epidemic on import and export trade. The estimated results are shown in Table 5.

As can be seen from the estimated results in Table 5, in both groups, the epidemic disease has a significant negative impact on the trade growth of a country, while it has a significant negative impact on the trade ratio of a big country, which is consistent with the previous estimated results. In large country groups, the impact of epidemic duration is also consistent with the original estimates. This indicates that for large countries, the impact of pandemic disease is more likely to have a long-term trade diversion effect, while for small countries, the impact of pandemic disease is mainly manifested as a short-term suspension effect. Non-pandemic diseases have a positive impact on the trade growth of large countries, indicating that non-pandemic diseases do not have a strong impact on trade activities, but a small increase in trade due to the purchase of related medical supplies (such as imported drugs and influenza vaccines).In addition, the estimation results of other control variables are basically consistent with the original baseline estimation results, which proves that the original baseline estimation results have a certain robustness.

**Table 5**. Group estimation results: large and small countries.

|  | Model II Great Power | Model II Small Countries | Model Ⅳ Big Country | Model Ⅳ Small Countries |
| --- | --- | --- | --- | --- |
|  | *TradeGrowth* | *TradeGrowth* | *TradeRatio* | *TradeRatio* |
| *EDI* | 0.0000137^***^ | 0.0000153^*^ | 0.000000664^***^ | 3.33 e-10 |
|  | (4.39) | (2.54) | (5.12) | (0.21) |
| *NEDI* | 0.00723^*^ | 0.00487 | 0.0000650 | 0.00000366 |
|  | (2.50) | (0.57) | (0.54) | (1.62) |
| *EDITime* | 0.0285^***^ | 0.0127 | 0.00192^***^ | 0.0000104^*^ |
|  | (4.12) | (0.81) | (6.68) | (2.53) |
| *EDITime^2^* | 0.00326^***^ | 0.000224 | 0.000132^***^ | 0.000000945 |
|  | (4.28) | (0.07) | (4.15) | (1.20) |
| *NEDITime* | 0.0248 | 0.0117 | 0.00218^**^ | 0.0000278 |
|  | (1.41) | (0.13) | (2.97) | (1.21) |
| *NEDITime^2^* | 0.00930^*^ | 0.00334 | 0.000218 | 0.0000194 |
|  | (2.46) | (0.06) | (1.38) | (1.42) |
| Variable of control | control | control | control | control |
| Term of constant | 0.820^***^ | 1.058^***^ | 0.0161^***^ | 0.000120^***^ |
|  | (18.20) | (28.84) | (8.66) | (12.43) |
| Number of samples | 1265 | 1265 | 1265 | 1265 |

Note:^*^ *p < 0.05,*^**^ *p < 0.01,*^***^ *p < 0.001;*The values in parentheses are t-values.

6.2 Comparison of the impact of epidemics on trade between agricultural and non-agricultural countries

Since industrial production activities are more susceptible to the epidemic than agricultural production activities, the trade impact of the epidemic impact on non-agricultural countries may be stronger than that on agricultural countries. We attempted to observe whether such structural differences exist by further grouping regression. We used the ratio of agricultural added value to GDP published by the World Bank in 2018 as the standard to measure agricultural and non-agricultural countries. Countries with a ratio of more than 10% were defined as the agricultural group, while those with a ratio of less than 10% were defined as the non-agricultural group, and then grouped the estimates for comparative analysis. The estimated results are shown in Table 6.

According to the estimated results in Table 6, in the two groups, the epidemic disease has a significant negative impact on trade growth and trade ratio, which is consistent with the previous estimated results. By further comparing the absolute value of the regression coefficient, we can see that the impact of epidemic diseases on the trade growth of agricultural countries is slightly less than that of non-agricultural countries, which is in line with the theoretical expectation mentioned above.

Non-epidemic diseases have a positive and significant impact on the trade ratio of agricultural countries, a positive and significant impact on the trade growth of non-agricultural countries, and a negative and significant impact on the trade ratio of non-agricultural countries. The effect of non-epidemic duration on agricultural and non-agricultural countries is also reversed. This indicates that not only epidemic diseases have a greater impact on industrial production in general, but also non-epidemic diseases have a stronger impact on industrial production than agricultural production. Therefore, in the face of epidemic diseases or non-epidemic diseases, non-agricultural countries are more vulnerable to trade impacts than agricultural countries, resulting in long-term trade diversion effect.

**Table 6**. Group estimation results: agricultural countries and non-agricultural countries.

|  | Model II Agricultural country | Model II Non-agricultural countries | Model Ⅳ Agricultural country | Model Ⅳ Non-agricultural countries |
| --- | --- | --- | --- | --- |
|  | *TradeGrowth* | *TradeGrowth* | *TradeRatio* | *TradeRatio* |
| *EDI* | 0.0000149^**^ | 0.0000161^***^ | 0.000000466^***^ | 0.000000425^***^ |
|  | (3.18) | (3.76) | (3.67) | (3.89) |
| *NEDI* | 0.000720 | 0.00909^**^ | 0.000541^***^ | 0.000224^**^ |
|  | (0.12) | (2.83) | (3.45) | (2.74) |
| *EDITime* | 0.0255^**^ | 0.0183 | 0.00137^***^ | 0.00133^**^ |
|  | (3.20) | (1.05) | (6.37) | (3.00) |
| *EDITime^2^* | 0.00289^**^ | 0.00176 | 0.0000764^**^ | 0.000235^*^ |
|  | (3.19) | (0.43) | (3.12) | (2.26) |
| *NEDITime* | 0.00468 | 0.0506 | 0.00388^***^ | 0.00220^**^ |
|  | (0.17) | (1.69) | (5.31) | (2.88) |
| *NEDITime^2^* | 0.00665 | 0.00996 | 0.000547^***^ | 0.00122^***^ |
|  | (1.27) | (0.89) | (3.87) | (4.31) |
| Variable of control | control | control | control | control |
| Term of constant | 1.101^***^ | 0.844^***^ | 0.00801^***^ | 0.00599^***^ |
|  | (24.71) | (24.96) | (6.69) | (6.99) |
| Number of samples | 1334 | 1196 | 1334 | 1196 |

Note:^*^ *p < 0.05,*^**^ *p < 0.01,*^***^ *p < 0.001;*The values in parentheses are t-values.

6.3 Comparison of the impact of epidemics on trade between industrial and service countries

Similarly, we use the ratios of industrial added value to GDP and service added value to GDP published by the World Bank in 2018 as the standards to measure industrial and service countries, and define those countries with a ratio greater than 25% as the industrial and service group respectively, and then make estimation and comparative analysis in each group. The estimated results are shown in Table 7.

As can be seen from the estimated results in Table 7, the epidemic has a significant negative impact on trade growth and trade ratio in both the industrial group and the service group. The impact of epidemic duration was consistent with the original estimates. By further observing the absolute value of the regression coefficient, we can see that the impact on the trade of industrial countries is slightly less than that on the trade of service countries, but industrial countries are more vulnerable to the long-term impact of the epidemic to form a long-term trade transfer effect, or it is more difficult for industrial countries to recover their trade after the epidemic is over than that of service countries. In recent years, the proportion of service trade in global trade has been increasing. Service countries with high-tech, knowledge products and emerging service industries as the main players have lower substitutability of their dominant industries. Even if there is a more serious short-term trade contraction, there will be no significant industrial outflow phenomenon, and the corresponding trade share can still be recovered after the end of the epidemic. There will be no long-term trade diversion effect; However, countries that mainly export industrial products are often less developed countries, and the growth of service trade is slow. Even if they can survive a certain trade contraction in the short term due to inventory protection, they are more likely to form industrial outflow in the long term trade contraction, and thus form long-term trade transfer and lose market share. This also shows that when the epidemic occurs, we should pay special attention to the outflow of industrial production, especially the middle and low-end manufacturing industry, increase investment, enhance the technological growth in industrial production, improve the added value of Chinese industrial production in the international value chain, reduce the substitutability of export products, and ensure the stable development of industrial structure.

Non-epidemic diseases have a positive effect on the trade ratio of industrial countries and a negative effect on the trade ratio of service countries. The effect of the duration of non-pandemic diseases on the trade ratios of industrial and service countries is also reversed. This suggests that non-pandemic diseases, such as common influenza, do not halt industrial activity, but may shrink service activity.

In conclusion, both epidemic and non-epidemic diseases have a greater impact on service countries in the short term. In the medium to long term, the impact of epidemic diseases on industrial countries is greater, while the impact of non-epidemic diseases on service countries is still greater.

**Table 7.** Group estimation results: industrial countries and service countries.

|  | Model II Industrial countries | Model II Service country | Model Ⅳ Industrial countries | Model Ⅳ Service countries |
| --- | --- | --- | --- | --- |
|  | *TradeGrowth* | *TradeGrowth* | *TradeRatio* | *TradeRatio* |
| *EDI* | 0.0000130^***^ | 0.0000152^**^ | 0.000000558^***^ | 0.000000371^***^ |
|  | (3.51) | (3.17) | (4.54) | (4.34) |
| *NEDI* | 0.00477 | 0.00673 | 0.000299^**^ | 0.000505^***^ |
|  | (1.38) | (1.21) | (2.60) | (5.12) |
| *EDITime* | 0.0309^***^ | 0.00917 | 0.00190^***^ | 0.000762^**^ |
|  | (3.87) | (0.68) | (7.18) | (3.16) |
| *EDITime^2^* | 0.00334^***^ | 0.000390 | 0.000115^***^ | 0.000107^*^ |
|  | (3.96) | (0.16) | (4.10) | (2.41) |
| *NEDITime* | 0.00239 | 0.0630 | 0.00325^***^ | 0.00305^***^ |
|  | (0.11) | (1.65) | (4.44) | (4.48) |
| *NEDITime^2^* | 0.00600 | 0.0118 | 0.000457^**^ | 0.00141^***^ |
|  | (1.37) | (0.86) | (3.13) | (5.81) |
| Variable of control | control | control | control | control |
| Term of constant | 0.908^***^ | 1.038^***^ | 0.0111^***^ | 0.00259^***^ |
|  | (22.13) | (26.98) | (8.17) | (3.78) |
| Number of samples | 1311 | 1219 | 1311 | 1219 |

Note:^*^ *p < 0.05,*^**^ *p < 0.01,*^***^ *p < 0.001;*The values in parentheses are t-values.

6.4 Summary of structural analysis and robustness test

To sum up, the original baseline estimation results are relatively robust. In addition, through the analysis of the new grouping structure, the following conclusions are further drawn: the impact of epidemics on the trade of big countries is greater than that of small countries, and the impact of epidemics on the trade of big countries is more likely to form a long-term trade diversion effect. The impact of epidemic disease on the trade of agricultural countries is less than that of non-agricultural countries, and non-agricultural countries are more likely to form a long-term trade transfer effect under the impact of epidemic disease. In the short term, the trade impact of the epidemic on industrial countries is less than that on service countries. In the medium and long term, industrial countries are more likely to have a long-term trade diversion effect than service countries. These conclusions indicate that although China has achieved great results in dealing with the current COVID-19 pandemic, the adverse impact of future pandemic outbreaks or on China's industrial structure should not be taken lightly based on the possibility of virus mutation, the heterogeneity of epidemic outbreaks and the timeliness of vaccine development. In particular, China is both an economic and industrial power, and its share of trade in services is still much lower than that of developed countries. Therefore, it is necessary to adopt pro-industrial policies to ensure the stability, transformation and upgrading of China's industrial structure in the long run of the epidemic.

7. Conclusions and Policy Recommendations

Using balanced panel data of disease information and macro performance from 110 member countries of the World Health Organization (WHO) from 1996 to 2018, this paper examines the impact of disease shocks on trade and whether there is a long-term trade diversion effect, and further discusses the differences in the impact of epidemic and non-epidemic diseases on different countries. First, we sorted out the mechanism and path of the impact of the epidemic on trade and the long-term trade transfer effect, formed the theoretical framework of this paper, and put forward two empirical hypotheses on this basis. Then, we used the fixed-effect panel model to estimate. The results show that: first, the epidemic disease impact can have a negative impact on a country's trade growth and ratio and has a long-term trade transfer effect, while the non-epidemic disease impact does not have a robust significant impact. Second, there is a negative concave nonlinear effect between the duration of epidemic disease and trade impact, indicating that with the increasing duration of epidemic disease, the probability of long-term trade diversion effect is also increasing. Then, based on the heterogeneity of countries, we made a group estimation to carry out a comparative analysis, and reached three conclusions: first, the impact of the epidemic on the trade of big countries was greater than that of small countries, and the impact of the epidemic on big countries was more likely to form a long-term trade diversion effect. Secondly, the impact of epidemic disease on the trade of agricultural countries is less than that of non-agricultural countries, and non-agricultural countries are more likely to form a long-term trade transfer effect under the impact of epidemic disease. Third, in the short term, the trade impact of the epidemic on industrial countries is less than that on service countries. In the medium and long term, industrial countries are more likely to have a long-term trade diversion effect than service countries. At the same time, grouping estimation also proves that the original estimation results have good robustness.

This paper comprehensively reviews the theoretical mechanism of the impact of epidemic disease on trade and the formation of long-term trade transfer effect, and obtains good robustness estimation results, which can provide supplementary research literature and general empirical evidence for whether epidemic disease will impact a country's trade and the formation of long-term trade transfer effect. According to the conclusion of this study, the impact of epidemic disease has a long-term trade transfer effect, which means that the impact of epidemic disease has a deep impact on the industrial structure of a country, and may affect the stability of a country's long-term economic growth. The recurrence of COVID-19 in the autumn and winter of 2020 prompted countries around the world to strengthen epidemic prevention and control efforts, but other countries except China have yet to recover their pre-pandemic economic and trade growth in the post-pandemic era. In April 2021, the International Monetary Fund proposed in its World Economic Outlook that many countries will not recover their pre-pandemic GDP growth levels until 2023, indicating that the general impact of COVID-19 on countries' economic and industrial structures may have a long-lasting impact. China's experience of achieving positive economic and trade growth in a short period of time also provides a blueprint for other countries' foreign trade recovery. To effectively address the continued impact of the epidemic on the economy and trade, countries not only need to step up macro policy implementation, but also need to strengthen international communication and deepen international cooperation. This includes: First, we should expand economic diplomacy, advocate free trade and multilateral trade, oppose trade protectionism, and gradually resume trade exchanges (especially expanding trade in epidemic prevention and quarantine materials and equipment) while raising trade health standards, so as to give play to the role of trade in promoting economic recovery. Second, we need to accelerate the building of a community with a shared future for mankind, advocate an international political and economic order featuring mutually beneficial cooperation and win-win development, and jointly address the impact of the epidemic on industries and the economy.

The continuous growth of foreign trade has been one of the main force of economic growth of our country. It is particularly important for China's steady growth, steady employment, deleveraging, structural adjustment and transformation and upgrading to avoid a large shrinkage of foreign trade, especially a long-term trade transfer impact caused by epidemic disease. As far as possible, we should fully consider that the occurrence of public health emergencies and the deterioration of external environment rotation will bring adverse effects on the trade chain structure in the medium and long term, and then adopt a series of industry-specific support policies in a proactive manner.The research on the impact of the epidemic on a country's trade is also worth continuing to follow up and carry out a deeper and more comprehensive micro demonstration.

**Funding:** This research was funded by "Globalization and Regionalization: A Study on the Law and Trend of the Development of International Economic and Trade Organizations" (Project No. 3162021ZK01),and by Hebei Provincial Department of education science research plan project major research project of Humanities and Social Sciences "Research on the promotion path of industrial chain of Hebei coastal counties (districts and cities) from the perspective of coordinated development of Beijing, Tianjin and Hebei" (Grant No. ZD202104) “, and by the Annual project of Hebei Social Science Foundation "Research on the development history of Hebei coastal cities" (No.: HB18WH06).

**Institutional Review Board Statement:** Not applicable.

**Informed Consent Statement:** Not applicable.

**Data Availability Statement:** The data used to support the findings of this study are available from the corresponding author upon request.

**Conflicts of Interest:** We declare that we do not have any commercial or associative interest that represents a conflict of interest in connection with the work submitted.

References

1. Cao Liang, Jiang Hongbin, Chen Xiaohong. Research on the trade creation and diversion effect of CAFTA . Macroeconomic Research, 2013(06) : 29-34+71.
2. Chen Hanlin. Analysis and countermeasures of the trade transfer effect of US anti-dumping against China . International Trade, 2008, (09) : 18-22.
3. Guo Yan, Li Xiubin. The Increasingly Prominent Effect of Trade Diversion: A Study on the Change of the import trade pattern of Textiles and Apparel in the United States in the era of zero quota . International Trade, 2004, (07) : 37-40.
4. Hao Liang. Manufacturer decision order, trade diversion effect and determination of optimal anti-dumping duty . Issues of International Trade, 2017, (05) : 139-152.
5. Huang Yueheng, Luo Zhengjing, Feng Zongxian. On endogenous technical barriers to trade in commodity quarantine . Journal of Quantitative & Technical Economics, 2004, (05) : 25-30.
6. Huang Zhaoyin. SARS challenges China's foreign trade . Journal of Wuhan University (Social Science Edition), 2003, (04) : 408-409.
7. Liu Hongguang, Zhang Jie, Zhu Zhongxiang, Zhang Zimeng. Quantitative analysis of global industrial trade transfer before and after financial crisis . Economic Geography, 2019,39 (01) : 96-103. (in Chinese)
8. Liu G Yi, Cao Jie. The Trade Diversion Effect of EU Anti-dumping against China: An Empirical Analysis Based on Product Perspective . International Trade Review, 2011, (07) : 91-101.]
9. Liu Graviti, Shao Min. The Trade Diversion Effect of Indian Anti-dumping against China: An Empirical Analysis Based on Product Perspective . International Economic and Trade Exploration, 2009,25 (09) : 48-53.
10. Shao Bai, Wei Shan, Liu Mingjie. Study on the impact and Countermeasures of International Public health Emergencies on international trade . Port Health Control, 2019,24 (04) : 1-3.
11. Shen Hao, Yang Yong. Analysis on the trade creation and trade diversion effect of African economic integration . International Trade Issues, 2008, (04) : 49-54.
12. Shi Changhua, Gong Hanying. Preliminary study on improving the system of technical trade measures in response to public health emergencies in China . Port Health Control, 2013,18 (03) : 1-4.
13. Sun Ming, Yang Shihui. Trade diversion Effects of Countervailing Claims: An empirical analysis based on panel Data . Finance and Trade Research, 2011,22 (05) : 65-70.
14. Xiang Hongjin. Study on the trade restriction Effect and trade diversion Effect of Foreign Anti-dumping Measures against China . Journal of Quantitative & Technical Economics, 2008,25 (10) : 75-86.
15. Xu Fen, Liu Hongman. Study on the trade creation and trade diversion Effect of China's agricultural imports in Free Trade Zone: Based on SYSGMM Estimation of import demand model . Issues of Agricultural Economy, 2017,38 (09) : 76-84+111-112.
16. Zhang Bin, Zhang Shu. Trade Creation and Transfer of the United States in NAFTA: 1994~2003. World Economy, 2005, (08) : 13-23.
17. Analysis of the trade diversion effect of the U.S. anti-dumping Complaint . International Economic and Trade Exploration, 2013,29 (04) : 16-26.
18. Anderson T. W.，Cheng H.. Formulation and Estimation of Dynamic Models Using Panel Data. Journal of Econometrics，1982，18(1)：47-82.
19. Bai J. Panel Data Models With Interactive Fixed Effects. Econometrica，2009，77(4)：1229-1279.
20. Baylis K.，Perloff J. M. Trade Diversion from Tomato Suspension Agreements. Canadian Journal of Economics/Revue Canadienne D'économique，2010，43(1)：127-151.
21. Bohara A. K.，Gawande K.，Sanguinetti P. Trade Diversion and Declining Tariffs：Evidence from Mercosur. Journal of International Economics，2004，64(1)：65-88.
22. Brenton P. Anti-dumping Policies in the EU and Trade Diversion. European Journal of Political Economy，2001，17(3)：593-607.
23. Cheong J.，Kwak D. W.，Tang K. K. Can Trade Agreements Curtail Trade Creation and Prevent Trade Diversion?. Review of International Economics，2015，23(2)：221-238.
24. Clausing K. A. Trade Creation and Trade Diversion in the Canada–United States Free Trade Agreement. Canadian Journal of Economics/Revue Canadienne D'économique，2001，34(3)：677-696.
25. Dai M.，Yotov Y. V.，Zylkin T. On the Trade-diversion Effects of Free Trade Agreements. Economics Letters，2014，122(2)：321-325.
26. Datta A.，Kouliavtsev M. NAFTA and the Realignment of Textile and Apparel Trade：Trade Creation or Trade Diversion?. Review of International Economics，2009，17(1)：172-186.
27. Headey D. Rethinking the Global Food Crisis：The Role of Trade Shocks. Food Policy，2011，36(2)： 136-146.
28. Leadon D. P.，Herholz C. Globalisation of Trade and the Spread of Infectious Disease. Equine vet. Educ. Manual，2009，8.
29. Magee C. S. P. New Measures of Trade Creation and Trade Diversion. Journal of International Economics，2008，75(2)：349-362.
30. Magee C. S. P. Trade Creation，Trade Diversion，and the General Equilibrium Effects of Regional Trade Agreements：A Study of the European Community–Turkey Customs Union. Review of World Economics，2016，152(2)：383-399.
31. Park S. The Trade Depressing and Trade Diversion Effects of Antidumping Actions：The Case of China. China Economic Review，2009，20(3)：542-548.
32. Perrings C.，Levin S.，Daszak P. The Economics of Infectious Disease，Trade and Pandemic Risk. 2018.
33. Sun L.，Reed M. R. Impacts of Free Trade Agreements on Agricultural Trade Creation and Trade Diversion. American Journal of Agricultural Economics，2010，92(5)：1351-1363.
34. Yang S.，Martinez-Zarzoso I. A Panel Data Analysis of Trade Creation and Trade Diversion Effects：The Case of ASEAN–China Free Trade Area. China Economic Review，2014，29：138-151.
