# Supplementary material for: Trade shocks and trade diversion due to epidemic diseases: Evidence from 110 countries
Source: PLoS One. 2024 May 31;19(5):e0301828. doi: 10.1371/journal.pone.0301828 (PMC11142499; doi:10.1371/journal.pone.0301828)
Supplement: Attachment — Submitted filename: Response to Reviewers.doc [file pone.0301828.s003.doc]

**Response to Reviewers**

**Respected Editors and Reviewers,**

Thanks for your patient reviewing and lots of great suggestions which would make this paper much better. Refer to the suggestions, we conduct very careful and detailed revision according to every great suggestion, which could be summarized to seven major revisions as follow:

1. First, we rearrange the abstract and introduction, to focus on the purpose, methodology, findings and practical implications of this paper. We also revise many critical parts especially the theoretical framework, variable illustration and empirical results discussion, so that this contents could be more precise and clear.
2. Second, we modify the diverse terminology, in the new manuscript we only use trade shock to express the short-term impact which can be recovered after epidemics, and only use trade diversion to express the long-term impact which cannot be recovered even after epidemics. We also revise the other problems with diverse terminology, so that the content could be more focused while reading.
3. Third, we update the reference literatures, especially those published in recent three years. Thanks for your suggestion, we found many great literatures could be very good supplements to enhance several assumption and stylized facts discuss in this research.
4. Fourth, we fatherly conduct the VIF test after baseline estimation to explain the reason why Multicollinearity can be ignored in this paper. Also in the panel estimation there was no variable omitted and the effectiveness of the estimation was still available. Actually, due to that the epidemic disease mostly occurred in a sudden way, often made it an endogenous variable without definite relation to the macroeconomic variables. We think the Multicollinearity problem would not harm the results of this research.
5. We recheck the estimation results and updated the negative expressions. Sorry for that the symbol “-” was lost in the last manuscript. We also adjust the form including titles, subtitles, figs, tables, references etc. according to the journal’s requirements.
6. We keep and modify the results discussion of the comparison between the coefficients even the gap is very small as the reviewer’s question. We considered that small gap still might identify even few implication especially refereeing to the usual observing facts of the realities. However, we do agree with the reviewer’s suggestion. Macro data could also be influence by lots of un-control issues. We modified the expression, turn the view into a discussion of possibility, so that there would be better communication with the readers.
7. Final, we revise the English writing of the whole paper due to help from our native speaker friends. We also erase the unnecessary contents and exclude all of the first-person references throughout the paper so that it could achieve the requirements.

Thanks so much that your comprehensive consideration help us to improve this paper a lot. However, the limitation of this research is that the COVID-19 is not included in the samples, and updating the samples to the year of 2022 is also one of suggestion from the reviewers. The reason why we could not include the samples after 2019 and COVID-19 is due to two challenges. First, the COVID-19 is so different from all of the epidemics during the last two decades with its such a worldwide impact range, the macroeconomic variable became greatly interrelated so that the cross-sectional or panel identification during this three years is not available. Meanwhile, this paper aimed to show to what extent the endogenous impact such as epidemics could impact the trade and reshape the industrial structure via trade shock and trade diversion. These can only be identified when the total trade is still in the normal growth path while some trade disappear. During the COVID-19, the global trade decrease jointly, also made the identification of trade shock or diversion for specific countries not available any more. Second, during last three years, the whole world was fighting with the COVID-19, let alone the WHO. The usual regional epidemics data has been covered by the COVID-19 and WHO has no such a huge team to update the tracing of original epidemics, and also governmental statistics has to focus on COVID-19 and chose to ignore the original epidemics when serious rapid spreading occurs, which make the dataset not continuous if involved samples after 2019. Considering this two practical restrictions, we choose the samples during 1996-2018 to complete this research. Even though, we still think the results of this paper could explain the trade shock and diversion impacted by epidemics, and bring some implication for us to understand or foresee what would happen to the global trade and industrial structure in the next several years. It is believed that after a few years, when we look back, there could be many methods to conduct the analysis of COVID-19 such as take the historical phase as a worldwide random natural experiment or find fantastic breakpoints. In this paper, the results shows that even very few fatal threats could leads to panic and harm the original trade activities. With such a serious influence from COVID-19, we believed that the forward-looking policies and enhancing international economic cooperation should be implemented rapidly while the epidemic turns to Omikjon.

We cannot illustrate every detailed modification in this response, please find all of the revisions in the new manuscript. Great thanks to the editors and reviewers! Without your kind consideration, this paper could not be improved so fast. Looking forward to your further consideration and great suggestion!

Best regards,

All authors of this paper

2023-2-4
