# Supplementary material for: Trade shocks and trade diversion due to epidemic diseases: Evidence from 110 countries
Source: PLoS One. 2024 May 31;19(5):e0301828. doi: 10.1371/journal.pone.0301828 (PMC11142499; doi:10.1371/journal.pone.0301828)
Supplement: Attachment — Submitted filename: PONE-D-22-34151R1-0510.doc [file pone.0301828.s006.doc]

Article

***Trade Shocks and Trade Diversion of Epidemic Diseases: Evidence from 110 Countries***

**Naixi Liu ^1&^ ,Yu Li ^2&^, Mingzhe Jiang ^1&^ , Bangfan Liu^3,4^*^#^ and Chuanhong Song^5^*^#^**

^1^ School of International Economics, China Foreign Affairs University, Beijing, , China

^2^ College of Economics and Management, China Agricultural University, Beijing

^3^ School of public administration Yanshan University, Qinhuangdao ,China

^4^ Hebei public policy evaluation and Research Center, Qinhuangdao ,China

^5^ The Center For Economic Research,Shandong University, Jinan, China

***** Corresponding author: liubangfan@ysu.edu.cn(B.L); mwgtgzy@126.com (C.S.)

& These authors contributed equally to this work.

\# These authors also contributed equally to this work.

**Abstract**

The COVID-19 has greatly shocked the global trade, made the latest years a very special phase. However, even during the normal time, epidemic disease was shocking the trade of specific countries, just not such a wide range. Some of the trade shocks can be recovered after the disease transmission is over, however some could be not, which means sometimes the specific countries would lose its original trade ratio due to trade diversion, or say epidemic disease could lead to unexpected industry restructuring. To identify this challenge, based on the data of 110 WHO members from 1996 to 2018, we use a fixed effect panel model supported by Hausman Test, to empirically identify whether epidemic diseases would cause trade shocks and trade diversion. It is found that: firstly, epidemic disease can lead to negative shocks on a country's trade growth and its ratio of the worldwide trade; secondly, with a longer duration of epidemic diseases, the probability of trade diversion effect is increasing. We use further estimation based on country heterogeneity to test the validation and robustness, our baseline mode was supported solidly. This provide quite a considerable concern as the shock of COVID-19 lasting, lots of countries is not just facing the problem of temporary trade shocks, but also the challenge of trade diversions, and its probability is increasing rapidly especially for the late-developed countries due to their lack of epidemic containing and vaccine producing capability. Even for the middle and high income countries, the global industry chain restructuring also cannot ignored, forward-looking policies should be implemented in advance, it would be too late when the long term trade damage shows.

**Keywords**

Epidemic Disease; Trade Shocks; Trade Diversion

1. Introduction

Since the outbreak of COVID-19, the global economy has been greatly shocked, whether the impact is caused by the COVID-19 or other reasons. With the threats decreasing, the economy is recovering. Will the trade shock due to COVID-19 disappear or leave a huge scar? COVID-19 made a great impression to worldwide range, however, showed by the WHO data, regional epidemic diseases were always existing somewhere in the world, and shocking the trade of specific counties even during the normal time. Sometimes the diseases impact a very short time or limited range, the trade shock was just temporally interrupted or suspended, and would recover after the transmission of disease (Fernandes et al., 2020)[1]. However, sometimes the trade partners will lose patience and awaiting ability due to long-term supply chain interruption, the employees and workers will lose confidence to returning their positions which urge them to find new living profession (Yu Zhen et al., 2020; Dai Chenghao, 2022)[2,3], the plants and machines will be depreciated due to major problem of long-term idle, such negative impacts would make the trade shocks un-recovered(Gruszczynski and Lukasz, 2020; Arnon et al., 2020)[4,5]. The countries suffering epidemic disease will face the unexpected industry restructuring due to trade shock and diversion(Handfield et al., 2020; Queiroz et al., 2020)[6,7], which make the epidemic disease a greatly bad exogenous impact for economies(White and Alexandre, 2020; Delivorias and Nicole, 2020; Eichenbaum et al., 2021)[8-10].

To what extent the epidemic disease will lead to trade shock and trade diversion? Will COVID-19 lead to worldwide industry restructuring due to its huge-rangeimpact? In general, there are two limitations in previous relevant studies, which make them unable to effectively answer the two important questions.

First, there are few empirical studies on the impact of epidemics on trade. Some scholars have studied the impact of epidemics on trade, such as the temporary stagnation of trade in the short term (Huang Yueheng et al., 2004; Leadon and Herholz, 2009; Shi Changhua and Gong Hanying, 2013)[11-13],however most of them just used qualitative discussiontrade diversion (Huang Zhaoyin, 2003; Shao Bai et al., 2019)[14,15]. Second, there are few empirical studies on the trade diversion caused by epidemics. Some scholars have discussed trade shock and trade diversion(Huang Zhaoyin, 2003;Leadon and Herholz, 2009;Shi Changhua and Gong Hanying, 2013;Perrings et al., 2018;Shao Bai et al., 2019)[14,12,13,16,15], and few have conducted studies on the trade diversion issue, especially empirical studies on trade diversion, which cannot provide relatively general argumentative and solid support to answer the questions we concern.

We innovatively uses the balanced panel data of disease records of 110 member countries released by the World Health Organization from 1996 to 2018 and their macro-data from World Bank and WTO, to make an empirical study on whether epidemic disease lead to trade shock and trade diversion. We did not use the data after and including 2019, because the COVID-19 is so different from the regional and limited epidemic disease in the past. COVID-19 impacted the worldwide economy due to its such a huge range which made it very special issue even in the human’s history(Kostova et al., 2019; Wanjala and Kevin, 2020; Brenton et al., 2020; Welfens and Paul, 2020; Megits et al., 2020; Antràs Pol et al., 2020)[17-22], also made the worldwide macro-data including and after 2019 a greatly interrelated set that cannot be identified by cross-sectional estimation. To achieve more precise identification, we only keep the data before 2019. However, our conclusion could also bring valuable implication to understand the influence of COVID-19. We also conducted the estimation based on country heterogeneity to test the validation and robustness, which illustrated our baseline model is supported solidly.

In essence, this paper studies the impact of infectious diseases on economic development, which is called "infectious disease economics". Epidemiological economics is a developing discipline, and the theory is not mature or systematic. Check the research literature at home and abroad, there are not many papers directly studying the economics of infectious diseases. In WOS, there are only a dozen papers directly on infectious diseases + economics, and only two of them directly discuss the relationship between infectious diseases and economic development. Dimitri Nicola points out:Epidemic, infectious, diseases affect a large number of individuals across developing as well as developed countries.[23] The article of Gong Mengchun etc. states: In the setting of epidemics of communicable diseases, early initiation of epidemiological and clinical data collection and analysis and conducting relevant researches are essential to the success of epidemic containment. The authors propose the evaluation methods for case reports, randomized controlled trials (RCTs), real-world evidence studies and health economics researches during an epidemic. The data from health economics research also provide important support for optimizing communicable disease prevention and control strategies. Herein we summarize the health economics research methods, study design, and technical points during the outbreaks. We recommend that clinical research and health economics research be incorporated into the prevention and control plan and measures be taken to ensure both the standards and feasibility of these studies to improve the response capacity against outbreaks of communicable diseases.[24]	In addition, Boucekkine,Raouf Carvajal, etc.,[25]and Sung Jane[26] also discuss the relationship between epidemics and economic development. There are only 28 literatures on the topic of epidemiological economics in China National Knowledge Network, among which 4 papers are directly titled "epidemiological economics", namely: Progress in research on Economic impact of pandemics and Intervention policies [27], Advances in theoretical and empirical research on epidemiological economics [28], Epidemiological economics and the Transformation of health policy [29], Pandemic Economics: the 1918 pandemic influenza and its Modern Significance [30]. The study of the impact of pandemics on trade shocks and trade diversion is undoubtedly a useful supplement to epidemiological economics.

The structure of this paper is as follow: the first part is the introduction; the second part is literature review; the third part is the theoretical framework, in which we elaborate the impact mechanism and path of epidemic disease that may lead to trade shock and diversion; the fourth part is model construction, data selection and variable interpretation; the fifth part is the estimation results and discussion; the sixth part is to further structural comparative estimation and robustness test based on country heterogeneity; the final part is the conclusions.

2. Literature review

2.1 Research on the impact of epidemics on trade shock and trade diversion

The World Health Organization's "Building a Secure Future: Global Public Health Security in the 21st Century" report notes that disease outbreaks can cause significant economic losses by disrupting trade and travel. Some scholars also studied the impact of epidemics on trade.

Huang Zhaoyin (2003)[14] analyzed the impact of SARS on China's trade and found that the trade fluctuation was small in the month of SARS outbreak. However, due to the lag, business activities were postponed or even cancelled in the following months, the export threshold of Chinese products was raised, the export share decreased, and foreign trade was difficult to carry out. Huang Yueheng et al. (2004)[11], based on the perspective of importing countries, linked the setting of technical trade barriers for different countries with the probability of epidemic occurrence in exporting countries, and raised the technical import threshold for countries with high probability of epidemic occurrence to reduce the risk of epidemic impact on domestic trade. Leadon and Herholz (2009)[12] illustrated the historical evidence of the introduction of pathogens into disease-free areas by the horse product trade, and strongly demonstrated that the disease caused the short-term stagnation of the horse trade. Shi Changhua and Gong Hanying (2013)[13] reviewed various international public health emergencies, and found that the health control measures taken by various countries for personnel, entry-exit goods and vehicles brought huge losses to various economies, especially foreign trade. Perrings et al. (2018)[16] believed that the emergence and spread of infectious diseases were closely related to the growth of trade network. When the epidemic occurred, the epidemic could quickly spread to all directions of the trade network through trade activities, making importers become the driving force in determining the trade flow, and the import activities avoided the affected countries. Shao Bai et al. (2019)[15] analyzed the impact of international public health emergencies on countries' trade: the interruption of foreign economic exchanges and the reduction or delayed transfer of foreign trade orders in the affected countries; Neighboring countries of the affected countries have seen a significant shift in trade, resulting in a substantial increase in export demand.

2.2 Other relevant impact on trade and formation of trade diversion

In general, in addition to the epidemic factor, there are many discussions on the impact of other factors on trade and the formation of trade diversion effect in previous literature. There are mainly three reasons.

2.2.1 Trade agreements and trade barriers

Clausing (2001)[31] analyzed the influence of the FTA on the trade patterns of the two countries, and the results showed that the FTA had a trade creation effect, but no trade diversion effect. Bohara et al. (2004)[32] verified the view that "trade integration may exacerbate inefficiency" and found that trade was indeed transferred to free trade area partner countries, but this trade diversion effect was internalized and weakened due to the disappearance of trade barriers. Guo Yan and Li Xiubin (2004)[33] studied the zero-quota and zero-tariff textile trade agreements signed by the United States with some developing countries on the basis of the North American Free Trade Agreement (NAFTA), and observed that the traditional textile exporting countries in Asia suffered losses from trade diversion. Zhang Bin and Zhang Shu (2005)[34] further analyzed the trade diversion of various commodities in the United States under the influence of NAFTA from 1994 to 2003, and found that there was an annual discontinuous trade diversion effect on labor-intensive manufactured goods. Magee (2008)[35] estimated the impact of regional trade agreements (Rtas) on trade flows with the econometric method, and the results showed that Rtas had a significant predictive signal effect and would continue to affect trade in the next 11 years. Shen Hao and Yang Yong (2008)[36] analyzed the trade diversion effect of African economic integration and found that the intra-community trade scale was too small to change the trade flow during 1999-2005. Datta and Kouliavtsev (2009)[37] studied the effects of wages, tariffs and exchange rates on the import structure of American textiles before and after the establishment of NAFTA, and the results showed that the trade creation effect was significant, while the trade diversion effect was not. Sun and Reed (2010)[38] conducted OLS estimation on the trade gravity model and calculated that there was a trade diversion effect among the members of the free trade Agreement, and agricultural trade increased substantially. Baylis and Perloff (2010)[39] found that when voluntary price constraints were strong, Mexico shifted its tomato export from the United States to Canada, while Canada increased its tomato product export to the United States. Cao Liang et al. (2013)[40] calculated the trade flow of China-Asean Free trade area by combining the trade gravity model, and demonstrated that there is no import trade diversion in China's bilateral relationship, but only trade creation. Yang and Martinez (2014)[41] also found that the trade agreement has brought huge trade creation to ASEAN. Dai et al. (2014)[42] collated historical data and came to the conclusion that free trade agreements transfer trade from non-member countries to member countries. Cheong et al. (2015)[43] explored the impact of preferential trade agreements on trade and observed that preferential trade agreements could weaken trade creation and shield the trade diversion effect of future trade agreements, thus minimizing inter-regional trade losses. Magee (2016)[44] estimated the general equilibrium effect of the European Community and found that the trade creation generated by the customs union was more than twice the trade diversion. Xu Fen and Liu Hongman (2017)[45] calculated that there was no trade diversion effect in China's agricultural exports to different free trade zones based on the trade gravity model and import demand model.

2.2.2 International trade disputes

Brenton (2001) [46]summarized the anti-dumping actions of various countries against the EU, and observed that the anti-dumping policy would lead to the transfer of the importing trade of the suing country to non-EU suppliers. Chen Hanlin (2008)[47] found that anti-dumping actions initiated by the United States against China greatly reduced China's exports to the United States, and part of the reduction may be transferred to the European Union. Xiang Hongjin (2008)[48] found that the export of similar products from South Korea, India and other countries to the United States increased in the same period. Liu Gravi and Shao Min (2009)[49] focused on Indian antidumping, listed the differences of Chinese product exports to India, and concluded that the transfer effect of Chinese product trade with comparative advantage was small. Park (2009)[50] focused on China's anti-dumping investigation, built a panel model and calculated that China's anti-dumping protection has significant trade inhibition effect and trade diversion effect. Sun Ming and Yang Shihui (2011)[51] took international countervailing cases from 1993 to 2007 as the research object and estimated that countervailing complaints caused the transfer of product imports from the accused country to the non-accused country. Liu Graviti and Cao Jie (2011)[52] started from the product categories of EU anti-dumping against China and found that the trade diversion effect was inconsistent with the intensity of anti-dumping, and then analyzed that the differences came from product competitiveness and product dependence. Zhang Yong (2013)[53] made use of the anti-dumping cases initiated by the United States from 1994 to 2008 to demonstrate that the complaint of the United States can only increase the import market share of the non-respondent country, without the trade diversion effect. Hao Liang (2017)[54] established an anti-dumping duty model and analyzed the optimal anti-dumping duties made by manufacturers at the same time or successively from the perspective of microeconomics, and calculated that there was no significant correlation between the level of the tax and the strength of the trade diversion effect.

2.2.3 Economic crisis

Headey(2011)[55] sorted out and observed the changes of international grain export volume and export price before the outbreak of the food crisis in 2008, and found that the surge of grain price lagged behind the rise of export volume, and the surge of food demand in the international market caused 15 to 27 food price changes worldwide, which then led to the change of a country's food import demand. Liu Hongguang et al.(2019)[56] sorted out the industrial trade diversion structure of major countries and regions before and after the 2008 financial crisis and argued that China was the largest net industrial transfer country, while the EU, the United States and other places gradually became the destinations of service trade diversion.

2.3 Summary of literatures

In conclusion, the conclusion of trade agreements (or changes in trade barriers), international trade disputes and economic crises can affect trade, while the conclusion of trade agreements and economic crises will lead to medium - and long-term trade diversion. However, in previous studies, there are relatively few empirical studies on whether epidemics can impact trade and cause long-term trade diversion effect. Only some scholars have conducted qualitative analysis on trade shock and trade diversion caused by specific epidemics, and no general conclusions can be drawn on this issue. This paper tries to provide a supplementary answer to this question.

3. Theoretical framework and hypothesis

We deconstruct and analyze the impact mechanism of epidemics on trade shock and trade diversion, establish a theoretical framework, and put forward two empirical hypotheses based on this. Firstly, for the trade without intra-industry or intra-multinationals trade, there are three impacts (as shown in Fig 1) :

Path ①: During the outbreak of epidemic disease, enterprises will spontaneously restrict production or stop work due to the panic and collective action to prevent and control the spread of epidemic disease. Foreign suppliers or downstream manufacturers will be forced to divert business to enterprises in other countries. The trade chain of affected countries will be interrupted, impacting foreign trade and forming short-term trade diversion. However, with the enclosure of the epidemic, enterprises in the affected countries tend to reduce their costs due to overcapacity(Kim Jaewook, 2020)[57], and the country's products and services will recover a certain degree of international competitiveness, thus slowing down the original trade diversion. However, affected by the shrinking demand during the epidemic, when production resumption causes overcapacity, it is difficult to realize such production resumption, and enterprises cannot recover their original international market share, resulting in long-term trade diversion.

Path ②: The outbreak of epidemic disease has caused "safety concerns" in the international market for products and services exported by the affected countries(Loayza, Norman, 2020; Aum Sangmin et al., 2021)[58,59]. For example, import restrictions or higher import standards have been imposed on products and services exported by the affected countries, which has impacted the export trade of the affected countries (for example, in the case of the outbreak of COVID-19 in 2020, South Korea, Indonesia and other countries have taken measures to restrict the import of agricultural products from China), the original trading partners have turned to trade with "safer countries", and the affected countries have been forced to suffer trade diversion. Moreover, to control the spreading of epidemic disease, commercial travel with the affected countries would also be restricted(Loayza, Norman, 2020)[58], so that the decrease of trade due to safety concerns would be worse.

Path ③ : With the increasing of the duration of the epidemic and the expansion of the affected countries, it is easy to form a rotation to prolong the time delay of the epidemic spreading. At this time, the impact on the trade will be expanding, and the probability of trade shock evolve into the trade diversion is also increasing.

**Figure 1.** Logical diagram of the impact of the disease on trade

Note: The arrows indicate the transmission mechanism of that the impact of epidemic diseases leads to trade shock and the trade diversion.

Secondly, for the intra-industry and intra-multinationals trade, when the worldwide industry is not in recession, it is more difficult to replace intra-industry and intra-multinational trade. Although when the epidemic occurs, trade shock will be sought and temporary trade diversion will be generated, and the possibility of long-term trade diversion will be relatively smaller after the epidemic ends. However, when the worldwide industry falls into recession, the trade of the intra-industry will remain at a low level for a long time, which will still impact the original trade chain and eventually form a long-term trade diversion.

Therefore, based on the above theoretical mechanism analysis, we propose two hypotheses to be tested:

Hypothesis 1: Epidemic diseases will lead to trade shock.

Hypothesis 2: Epidemic diseases will lead to trade diversion.

4. Data selection, model construction, and variable illustration

4.1 Data selection

In this paper, we use the disease data collected from WHO website releasing and news reports, and the macro economic data from World Bank and WTO database. After excluding the countries with serious data shortage, a balance panel data set of 110 countries during 1996-2018 were obtained as the estimation samples. The reason why we could not include the samples after 2019 and COVID-19 is due to two challenges. First, the COVID-19 is so different from all of the epidemics during the last two decades with its such a worldwide impact range(Kostova et al., 2019; Wanjala and Kevin, 2020; Brenton et al., 2020; Welfens and Paul, 2020; Megits et al., 2020; Antràs Pol et al., 2020)[17-22], the macroeconomic variable became greatly interrelated so that the cross-sectional or panel identification during this three years is not available. Meanwhile, this paper aimed to show to what extent the endogenous impact such as epidemics could impact the trade and reshape the industrial structure via trade shock and trade diversion. These can only be identified when the total trade is still in the normal growth path while some trade disappear. During the COVID-19, the global trade decrease jointly, also made the identification of trade shock or diversion for specific countries not available any more. Second, during last three years, the whole world was fighting with the COVID-19, let alone the WHO. The usual regional epidemics data has been covered by the COVID-19 and WHO has no such a huge team to update the tracing of original epidemics, and also governmental statistics has to focus on COVID-19 and chose to ignore the original epidemics when serious rapid spreading occurs, which make the dataset not continuous if involved samples after 2019. Considering this two practical restrictions, to achieve more precise identification, we choose the samples during 1996-2018 to complete this research.

4.2 Model construction

Even we have the discussion above, it is still critical to find a suitable method to identify the trade shock and trade diversion. In the past researches, especially in the dynamic research topics, most scholars used the first-order form of one variable to identify the shock or interrupt of its normal development, and use a ratio set of one variable to illustrate structure change of the system which the variable belongs to. This give us an easy way to capture the impact on trade from epidemic disease. We use the trade variables’ growth rate to identify whether the trade shock has occurred under the spreading of epidemic disease, and use the trade variables’ ratio of the world to identify whether the trade diversion has occurred while the epidemic disease lasting. According to the two empirical hypotheses proposed above and referring to the general panel estimation model setting, we set the model as follows:

Equation Ⅰ and Ⅱ for estimating the shock impact on export and overall trade due to the identification of growth dependent variable:

$\begin{matrix} ExportGrowth_{it}=\frac{Export_{it}}{Export_{i,t-1}}=\beta_{0}+\beta_{1}EDI_{it}+\beta_{2}NEDI_{it}+\beta_{3}EDITime_{it}+\beta_{4}EDITime_{it}^{2} \\ +\beta_{5}NEDITime_{it}+\beta_{6}NEDITime_{it}^{2}+\beta_{7}RTAIn_{it}+\beta_{8}Disputes_{it}+\sum_{k=9}^{m}\beta_{k}Z_{it}+\eta_{it} \end{matrix}$(Ⅰ)

$\begin{matrix} TradeGrowth_{it}=\frac{Trade_{it}}{Trade_{i,t-1}}=\beta_{0}+\beta_{1}EDI_{it}+\beta_{2}NEDI_{it}+\beta_{3}EDITime_{it}+\beta_{4}EDITime_{it}^{2} \\ +\beta_{5}NEDITime_{it}+\beta_{6}NEDITime_{it}^{2}+\beta_{7}RTAIn_{it}+\beta_{8}Disputes_{it}+\sum_{k=9}^{m}\beta_{k}Z_{it}+\gamma_{it} \end{matrix}$ (Ⅱ)

Equation Ⅲand Ⅳ for estimating the diversion impact on export and overall trade due to the identification of ratio dependent variable:

$\begin{matrix} ExportRatio_{it}=\frac{Export_{it}}{Export_{wt}}=\beta_{0}+\beta_{1}EDI_{it}+\beta_{2}NEDI_{it}+\beta_{3}EDITime_{it}+\beta_{4}EDITime_{it}^{2} \\ +\beta_{5}NEDITime_{it}+\beta_{6}NEDITime_{it}^{2}+\beta_{7}RTAIn_{it}+\sum_{k=8}^{m}\beta_{k}Z_{it}+\nu_{it} \end{matrix}$ (Ⅲ)

$\begin{matrix} TradeRatio_{it}=\frac{Trade_{it}}{Trade_{wt}}=\beta_{0}+\beta_{1}EDI_{it}+\beta_{2}NEDI_{it}+\beta_{3}EDITime_{it}+\beta_{4}EDITime_{it}^{2} \\ +\beta_{5}NEDITime_{it}+\beta_{6}NEDITime_{it}^{2}+\beta_{7}RTAIn_{it}+\sum_{k=8}^{m}\beta_{k}Z_{it}+\tau_{it} \end{matrix}$ (Ⅳ)

Where, i represents the country, t represents the corresponding year, and w represents the world total level.

The construction of the above model is mainly based on two considerations:

First, according to empirical hypothesis 1, we use the trade growth rate of a country in a certain year as the dependent variable of models I and II to estimate the shock impact of epidemic disease on a country's trade. This is because trade growth tends to have a certain inertia, and the impact of epidemics on a country's foreign trade is generally reflected in the impact on its original growth trend rather than the absolute value.

Secondly, according to empirical hypothesis 2, we use the ratio of a country's trade volume to the world's total trade volume in a certain year as the dependent variable of models Ⅲ and Ⅳ to estimate the trade diversion impact. This is because in a very short term such as in the nearest two years, it is difficult for the world trade structure to undergo greatly sudden changes. It is assumed that in the case of equilibrium without exogenous shock such as trade diversion impact, the trade volume of a country should keep growing while the total world trade volume increasing. The inconsistency between the growth of a country's trade volume and the world's total trade volume may be due to the fact that the country's original trade was replaced by the trade of other countries, or the country failed to enter the new expanding market. It is reasonable to believe that the change of the trade ratio of a country's total trade volume to the world's total trade volume can reflect the change of the country's trade structure.

4.3 Variable illustration

4.3.1 Dependent variables

ExportGrowthit And ExportRatioit Represents, respectively, the growth rate of exports and the ratio of exports to the world's total exportsit(The value of a country's trade, unit: USD million) and Exportwt(World exports, unit: million US dollars).

TradeGrowthit And TradeRatioit Respectively represents the growth rate of imports and exports and the ratio of imports and exports to the world's total imports and exports, by Tradeit(A country's Trade imports and exports, unit: million US dollars) and tradewt(World import and export, unit: millions of US dollars).

4.3.2 Independent Variables

EDIit: Epidemic Disease Impact represents the impact of epidemic diseases in country i in Year t. This variable is calculated using the sum of the case fatality rate of different epidemic diseases times the square value of the affected range (number of affected countries). We used the case fatality rate to reflect the economic impact of epidemic diseases, because when there is a probability of death, the social panic of epidemic diseases will be intensified, which will lead to spontaneous stagnation of economic activities, and make traders in affected countries voluntarily reduce their trade activities. The squared affected range is used because when multiple countries are hit by a pandemic, international trade tends to spiral downward and become more skewed towards only "safer countries", exacerbating trade shock and diversion. When there is no epidemic disease, the value of this variable would be 0.

NEDIit: Non-Epidemic Disease Impact represents the impact of non-epidemic diseases in country i in year t. This variable is calculated from the sum of the value of affected range (number of affected countries) times a 0-1 dummy variable to express whether there is non-epidemic disease in the specific country and year. The reason why we only weighted the number of affected countries without considering the fatality rate is because non-epidemic diseases are less contagious and less likely to cause widespread social panic. When there is no non-epidemic disease, the value of ths variable would be 0.

EDITimeit: Represents the duration of epidemic disease.The value of variable in the year of epidemic outbreak is 1, and increasing by 1 for each additional year of the duration thereafter. Considering the society and government will try all the best to control the disease spreading and people will get used to the disease threats, there might be a non-linear relationship between the duration of the outbreak and the effects of trade diversion, both the first and quadratic forms of EDITimeit are added to the estimation equation.

NEDITimeit: Represents the duration of non-epidemic disease.The value of the variable in the year of the outbreak of non-epidemic disease is 1, and increasing by 1 for each additional year of the duration thereafter. Similar to the consideration of EDITime, both the first and quadratic forms of NEDITimeit are added to the estimation equation.

4.3.3 Control Variables

RTAInit: Regional Trade Agreements Increase (Regional Trade Agreements Increase) is defined as the increase in the number of regional trade agreements signed by country i in year t. The data is from the WTO Trade agreement database, which is used to control the possible impact of new trade agreements signed by the country.

Disputesit: Defined as the number of cases in which Country i was the litigant in international trade disputes in year t. The number of trade disputes in all the years between the time a dispute was raised and its settlement was reached is added to the value of this variable. Considering that the impact of international trade disputes on a country's trade is mainly manifested as the impact on the change of trade growth rate in the current year (Chen Hanlin, 2008;Xiang Hongjin, 2008;Sun Ming and Yang Shihui, 2011)[47,48,51], but there is no inevitable causal logical connection with trade diversion (Zhang Yong, 2013;Hao Liang, 2017)[53,54]. Therefore, we only added international trade dispute control variables into models Ⅰ and Ⅱ.

Zit: is a group of control variables added by referring to previous research literatures, mainly to control other macroeconomic variables affecting trade changes, as shown in Table 1 below.

**Table 1.** Control variables -- macroeconomic variables

| **variable** | **meaning** | **Variable selection Description** |
| --- | --- | --- |
| ***GDPGrowth*** | GDP growth rate | Reflecting national income and national economic development, there is an endogenous relationship between GDP growth and trade growth of a country |
| ***POPGrowth*** | Population growth rate | Population growth drives demand growth, which in turn boosts import consumption |
| ***MGrowth*** | Broad money growth rate | Currency increase can reduce short-term nominal interest rate, but also make a country's currency relative depreciation, expand export trade and reduce import trade; and vice versa |
| ***R*** | Effective interest rate | The rise of real interest rate leads to capital inflow and relative currency appreciation, which expands import trade and reduces export trade, while the rise of savings under liquidity preference will expand trade surplus. And vice versa |
| ***Agriculture*** | Agricultural value added as a percentage of GDP | Reflecting the structure of the primary industry and influencing the export structure of agricultural products is particularly important for agricultural powers |
| ***Industry*** | Industrial added value as a percentage of GDP | To reflect the structure of the secondary industry and affect the export structure of industrial products is particularly important for industrial powers |

4.4.4 Endogeneity Problem

For more effective estimation, it is necessary to consider whether epidemics are endogenous variables that affect a country's trade growth rate and trade ratio. Actually, as a natural disaster that is difficult to predict in advance, epidemic disease is more likely to be seem as an endogenous variable in this paper. The expansion of the epidemics is often due to the cross-border and cross-regional movement of people but not the trade flow (Bontempi Elza et al., 2021)[60]. Therefore, the possible endogeneity of epidemic disease can be reasonably ignored.

5. Estimation results and discussion

5.1 Descriptive statistics of variables

The descriptive statistics of variables in the estimation equation are shown in Table 2.

**Table 2**. Statistical Table of variable description

| **variable** | **The mean** | **Standard deviation** | **Minimum value** | **Maximum value** |
| --- | --- | --- | --- | --- |
| ***ExportGrowth*** | 1.0923 | 0.2392 | 0.2941 | 4.7143 |
| ***TradeGrowth*** | 1.0842 | 0.1664 | 0.3581 | 1.9407 |
| ***ExportRatio*** | 0.0048 | 0.0144 | 3.50 e-07 | 0.1375 |
| ***TradeRatio*** | 0.0049 | 0.0156 | 1.85 e-06 | 0.1549 |
| ***EDI*** | 363.1312 | 1230.729 | 0 | 5854.32 |
| ***NEDI*** | 0.2217 | 1.3007 | 0 | 18 |
| ***EDITime*** | 0.4043 | 1.1682 | 0 | 14 |
| ***NEDITime*** | 0.0806 | 0.3894 | 0 | 7 |
| ***RTAIn*** | 0.3162 | 1.0346 | 0 | 28 |
| ***Disputes*** | 0.5711 | 2.1784 | 0 | 28 |
| ***GDPGrowth*** | 3.9657 | 4.2499 | 30.1451 | 88.9577 |
| ***POPGrowth*** | 1.5142 | 1.3285 | 9.0806 | 8.1179 |
| ***MGrowth*** | 17.4821 | 85.2695 | 99.8637 | 4105.573 |
| ***R*** | 7.3454 | 10.6454 | 93.5135 | 130.345 |
| ***Agriculture*** | 14.1097 | 12.3015 | 0.0249 | 79.0424 |
| ***Industry*** | 26.2827 | 11.2967 | 3.1502 | 74.1130 |

5.2 Estimation results and discussion

Due to significant country heterogeneity and macroeconomic cycle effects, country and year fixed effects should be controlled in economic logic. However, considering the possible partial sampling effect of sample data, we still need to test its measurement characteristics. The fixed effects model and random effects model were used to estimate the regression equation, and then the Hausmann test was used to make the selection judgment. The results show that (as shown in Table 3) it is more appropriate to select the fixed-effect model.

**Table 3**. Hausmann test results.

| **model** | **Ⅰ** | **Ⅱ** | **Ⅲ** | **Ⅳ** |
| --- | --- | --- | --- | --- |
| **χ^2^value** | 91.18^***^ | 124.96^***^ | 143.55^***^ | 145.87^***^ |
| **P values** | 0.000 | 0.000 | 0.000 | 0.000 |

Note:^*^ *p < 0.05,* ^**^*p < 0.01,* ^***^*p < 0.001;*The values in parentheses are t-values.

The macroeconomic variables used in our estimation all have first-order derivative properties (the discrete form is reflected in the growth rate or ratio), and since the number of estimated cross-section samples is much larger than the number of years, it is no longer necessary to conduct a separate unit root test (Anderson and Cheng, 1982; Bai, 2009)[61,62]. Further more

Therefore, we estimate the fixed effects of equations (I) to (IV) respectively, and the estimated results are shown in Table 4, which mainly include the following conclusions:

(1) Epidemic EDI had a significant negative impact on both ExportGrowth and TradeGrowth of a country's export trade, indicating that epidemic EDI could have an impact on a country's trade, and the empirical hypothesis was confirmed. The non-epidemic epidemic disease NEDI has no significant impact on the ExportGrowth and TradeGrowth of a country's export trade, indicating that the non-epidemic epidemic disease has no significant impact on a country's trade.

(2) EDI of epidemic disease has a significant negative impact on both ExportRatio and TradeRatio of a country's export to the world ratio and total import and export to the world ratio, indicating that epidemic disease impact has a long-term trade diversion effect, which is confirmed by the two empirical hypotheses. The influence of NEDI on the ExportRatio of a country's export volume and the exportratio of its total import and export volume in the world is not significant, indicating that the impact of NEDI does not have a long-term trade diversion effect.

(3) By observing the regression coefficient of epidemic duration EDITime, it can be found that the original form has a positive impact on trade growth and trade ratio, while the quadratic form has a negative impact on trade growth and trade ratio, indicating that the negative impact of epidemic duration on trade has a concave feature. This indicates that initially, with the stabilization of the epidemic spreading and the improvement of market expectation, especially the rapid economic activities recovery after a short-term epidemic, the impact of the epidemic on trade was alleviated. On the contrary, the demand of epidemic controlling facilities and health materials resulted in a small increase in the short-term trade. However, with the further increase of the duration of the epidemic, the confidence to overcome the epidemic has entered a new unstable state, and as the epidemic spreads to more countries, the external environment deteriorates, the short-term trade substitution gradually transitions to long-term trade diversion, and the impact of the epidemic on trade continues to intensify.

NEDI have a significant negative impact on the trade ratio, which may be due to the fact that NEDI have a small range of infection and cannot affect the external environment, while the short-term decline in their own trade makes the ratio decrease. Because of the relatively short duration of non-epidemic diseases, secondary forms do not show significant effects.

(4) RTAIn has a significant positive effect on Trade Growth of a country's import and export trade, which indicates that the growth of regional trade agreements brings about trade creation effect. RTAIn also has a significant positive impact on the Trade Ratio of a country's total import and export trade in the world, indicating that the trade creation effect brought by the growth of regional trade agreements is stronger than the trade substitution and crowding out effect, which is consistent with the conclusions of most previous studies.

(5) GDP growth rate, monetary growth rate and effective interest rate all have a significant impact on a country's trade growth, but have no significant impact on a country's trade to the world ratio. Among them, GDP growth can promote a country's trade growth, which is fully in line with our theoretical expectations when we select control variables. The rise of money growth will have a negative inhibitory effect on trade growth, because the demand response is faster and more flexible than the supply response. The short-term inhibitory effect of money growth on import trade is greater than the short-term promotion effect on export trade. The rise of real interest rate will also have a negative inhibitory effect on trade growth, indicating that the negative impact on trade through capital flow and exchange rate is greater than the positive impact on trade through investment and savings growth.

(6) The proportion of agricultural added value to GDP has a significant negative impact on a country's trade ratio. From a general point of view, the faster the export of agricultural products (natural resource-intensive products) is growing, the slower the export of industrial products is growing. In the past two decades, the proportion of agricultural trade in international trade has been declining, and the trade of industrial goods and services has taken an increasing share, which makes the proportion of trade of agricultural exporting countries in the world decreased.

(7) The ratio of industrial added value to GDP has a positive and significant impact on a country's trade growth, which indicates that the rise of a country's industrial output value can increase the export of industrial products, thus promoting the trade growth. Especially for those countries dominated by industrial exports (labor-intensive), this positive correlation is more consistent with the general fact.

**Table 4.** Estimation results table

|  | **Model I** | **Model II** | **Model Ⅲ** | **Model Ⅳ** |
| --- | --- | --- | --- | --- |
|  | *ExportGrowth* | *TradeGrowth* | *ExportRatio* | *TradeRatio* |
| ***EDI*** | -0.0000125^**^ | -0.0000150^***^ | -0.000000444^***^ | -0.000000428^***^ |
|  | (-2.82) | (-5.18) | (-5.26) | (-5.52) |
| ***NEDI*** | 0.00635 | 0.00570 | 0.000147 | 0.0000820 |
|  | (1.40) | (1.92) | (1.70) | (1.03) |
| ***EDITime*** | 0.0261^**^ | 0.0270^***^ | 0.00138^***^ | 0.00126^***^ |
|  | (2.68) | (4.22) | (7.41) | (7.37) |
| ***EDITime^2^*** | -0.00282^*^ | -0.00300^***^ | -0.0000780^***^ | -0.0000730^***^ |
|  | (-2.41) | (-3.91) | (-3.49) | (-3.55) |
| ***NEDITime*** | -0.0173 | -0.0318 | -0.00199^***^ | -0.00173^***^ |
|  | (-0.63) | (-1.78) | (-3.82) | (-3.61) |
| ***NEDITime^2^*** | 0.00721 | 0.0105^**^ | 0.000225 | 0.000169 |
|  | (1.18) | (2.61) | (1.93) | (1.58) |
| ***RTAIn*** | 0.00738 | 0.00759^*^ | 0.000161 | 0.000178^*^ |
|  | (1.59) | (2.49) | (1.87) | (2.25) |
| ***Disputes*** | -0.00210 | -0.00374 | - | - |
|  | (-0.58) | (-1.57) | - | - |
| ***GDPGrowth*** | 0.0214^***^ | 0.0181^***^ | 0.000000637 | 0.00000305 |
|  | (18.81) | (24.22) | (0.03) | (0.15) |
| ***POPGrowth*** | -0.00655 | -0.0102^*^ | -0.0000474 | -0.0000401 |
|  | (-0.96) | (-2.26) | (-0.36) | (-0.33) |
| ***MGrowth*** | -0.000103 | -0.0000740^*^ | -0.000000453 | -0.000000280 |
|  | (-1.86) | (-2.04) | (-0.43) | (-0.29) |
| ***R*** | -0.00530^***^ | -0.00436^***^ | -0.000000844 | 0.000000286 |
|  | (-9.85) | (-12.34) | (-0.08) | (0.03) |
| ***Agriculture*** | 0.000116 | -0.000209 | -0.0000961^***^ | -0.0000958^***^ |
|  | (0.10) | (-0.27) | (-4.25) | (-4.61) |
| ***Industry*** | 0.00302^*^ | 0.00350^***^ | -0.0000206 | -0.0000254 |
|  | (2.43) | (4.30) | (-0.87) | (-1.17) |
| **Term of constant** | 0.973^***^ | 0.971^***^ | 0.00651^***^ | 0.00679^***^ |
|  | (22.91) | (34.85) | (8.06) | (9.14) |
| **Number of samples** | 2530 | 2530 | 2530 | 2530 |

Note:^*^ *p < 0.05,*^**^ *p < 0.01,*^***^ *p < 0.001;*The values in parentheses are t-values.

As we know the multicollinearity would not influence the significance of our estimation above, and also in the fixed-effect panel estimation process, there is no variable omitted, which means the influence of multicollinearity could be very little. Even though, to exclude this influence more precisely, we take the variable EDI and control variables to conduct the VIF test, have the outcome as following table 5, shows that the probability of multicollinearity could be ingored. The estimation results and discussion above could be effective.

**Table 5.** VIF test results

| **Varibale** | **VIF** | **1/VIF** |
| --- | --- | --- |
| ***EDI*** | 1.75 | 0.571 |
| ***EDITime*** | 1.73 | 0.578538 |
| ***RTAIn*** | 1.08 | 0.928057 |
| ***Disputes*** | 1.06 | 0.941688 |
| ***GDPGrowth*** | 1.28 | 0.780812 |
| ***POPGrowth*** | 1.23 | 0.815188 |
| ***MGrowth*** | 1.26 | 0.793769 |
| ***R*** | 1.06 | 0.943815 |
| ***Agriculture*** | 1.57 | 0.638144 |
| ***Industry*** | 1.3 | 0.766417 |

6. Structural analysis and robustness test

In order to better understand the impact of the epidemic on different types of countries, we further carried out structural analysis and also as some robustness tests to some extent.

6.1 Comparison of the impact of epidemics on trade between large and small countries

For large countries, due to their dominant position in international trade, epidemics are more likely to have an impact on their trade. The complex industrial structure of large countries makes it more difficult to recover from the impact, which may lead to long-term trade diversion . For small countries, the impact of epidemics on trade growth is relatively small. We used the 2018 median GDP of each country published by the World Bank to classify large and small countries, and estimated the total impact of the epidemic on import and export trade. The estimated results are shown in Table 6.

As can be seen from the estimated results in Table 6, in both groups, the epidemic disease has a significant negative impact on the trade growth of a country, while it has a significant negative impact on the trade ratio of a big country, which is consistent with the previous estimated results. In large country groups, the impact of epidemic duration is also consistent with the original estimates. This indicates that for large countries, the impact of epidemic disease is more likely to have a long-term trade diversion, while for small countries, the impact of epidemic disease is mainly manifested as a short-term trade shock. Non-epidemic diseases have a positive impact on the trade growth of large countries, indicating that non-epidemic diseases do not have a strong impact on original trade activities, but a small increase in trade due to the purchase of related medical supplies (such as imported drugs and influenza vaccines). In addition, the estimation results of other control variables are basically consistent with the original baseline estimation results, which proves that the original baseline estimation results have a certain robustness.

**Table 6**. Group estimation results: large and small countries.

|  | **Model II Large Countries** | **Model II Small Countries** | **Model Ⅳ Large Country** | **Model Ⅳ Small Countries** |
| --- | --- | --- | --- | --- |
|  | *TradeGrowth* | *TradeGrowth* | *TradeRatio* | *TradeRatio* |
| ***EDI*** | -0.0000137^***^ | -0.0000153^*^ | -0.000000664^***^ | -3.33e-10 |
|  | (-4.39) | (-2.54) | (-5.12) | (-0.21) |
| ***NEDI*** | 0.00723^*^ | -0.00487 | 0.0000650 | 0.00000366 |
|  | (2.50) | (-0.57) | (0.54) | (1.62) |
| ***EDITime*** | 0.0285^***^ | 0.0127 | 0.00192^***^ | -0.0000104^*^ |
|  | (4.12) | (0.81) | (6.68) | (-2.53) |
| ***EDITime^2^*** | -0.00326^***^ | 0.000224 | -0.000132^***^ | 0.000000945 |
|  | (-4.28) | (0.07) | (-4.15) | (1.20) |
| ***NEDITime*** | -0.0248 | -0.0117 | -0.00218^**^ | -0.0000278 |
|  | (-1.41) | (-0.13) | (-2.97) | (-1.21) |
| ***NEDITime^2^*** | 0.00930^*^ | 0.00334 | 0.000218 | 0.0000194 |
|  | (2.46) | (0.06) | (1.38) | (1.42) |
| **Term of constant** | 0.820^***^ | 1.058^***^ | 0.0161^***^ | 0.000120^***^ |
|  | (18.20) | (28.84) | (8.66) | (12.43) |
| **Number of samples** | 1265 | 1265 | 1265 | 1265 |

Note:^*^ *p < 0.05,*^**^ *p < 0.01,*^***^ *p < 0.001;*The values in parentheses are t-values.

6.2 Comparison of the impact of epidemics on trade between agricultural and non-agricultural countries

Since the spreading velocity of epidemic disease, it highly related to the crowdedness of people and social contact activities, the industrial production activities especially manufacturing industry are more susceptible to the epidemic than agricultural producing activities, the trade impact of the epidemic impact on non-agricultural countries might be stronger than that on agricultural countries. We attempted to observe whether such structural differences exist by further grouping regression. We used the ratio of agricultural added value to GDP published by the World Bank in 2018 as the standard to measure agricultural and non-agricultural countries. Countries with a ratio of more than 10% were defined as the agricultural group, while those with a ratio of less than 10% were defined as the non-agricultural group, and then grouped the estimates for comparative analysis. The estimated results are shown in Table 7.

According to the estimated results in Table 6, in the two groups, the epidemic disease has a significant negative impact on trade growth and trade ratio, which is consistent with the previous estimated results. By further comparing the absolute value of the regression coefficient, we can see that the impact of epidemic diseases on the trade growth of agricultural countries is slightly less than that of non-agricultural countries.

Non-epidemic diseases have a positive and significant impact on the trade ratio of agricultural countries, a positive and significant impact on the trade growth of non-agricultural countries, and a negative and significant impact on the trade ratio of non-agricultural countries. The effect of non-epidemic duration on agricultural and non-agricultural countries is also reversed. This indicates that not only epidemic diseases have a greater impact on industrial production in general, but also non-epidemic diseases have a stronger impact on industrial production than agricultural production. Therefore, in the face of epidemic diseases or non-epidemic diseases, non-agricultural countries are more vulnerable to trade impacts than agricultural countries, resulting in long-term trade diversion effect.

**Table 7**. Group estimation results: agricultural countries and non-agricultural countries.

|  | **Model II Agricultural country** | **Model II Non-agricultural countries** | **Model Ⅳ Agricultural country** | **Model Ⅳ Non-agricultural countries** |
| --- | --- | --- | --- | --- |
|  | *TradeGrowth* | *TradeGrowth* | *TradeRatio* | *TradeRatio* |
| ***EDI*** | -0.0000149^**^ | -0.0000161^***^ | -0.000000466^***^ | -0.000000425^***^ |
|  | (-3.18) | (-3.76) | (-3.67) | (-3.89) |
| ***NEDI*** | -0.000720 | 0.00909^**^ | 0.000541^***^ | -0.000224^**^ |
|  | (-0.12) | (2.83) | (3.45) | (-2.74) |
| ***EDITime*** | 0.0255^**^ | 0.0183 | 0.00137^***^ | 0.00133^**^ |
|  | (3.20) | (1.05) | (6.37) | (3.00) |
| ***EDITime^2^*** | -0.00289^**^ | 0.00176 | -0.0000764^**^ | -0.000235^*^ |
|  | (-3.19) | (0.43) | (-3.12) | (-2.26) |
| ***NEDITime*** | -0.00468 | -0.0506 | -0.00388^***^ | 0.00220^**^ |
|  | (-0.17) | (-1.69) | (-5.31) | (2.88) |
| ***NEDITime^2^*** | 0.00665 | 0.00996 | 0.000547^***^ | -0.00122^***^ |
|  | (1.27) | (0.89) | (3.87) | (-4.31) |
|  |  |  |  |  |
| **Term of constant** | 1.101^***^ | 0.844^***^ | 0.00801^***^ | 0.00599^***^ |
|  | (24.71) | (24.96) | (6.69) | (6.99) |
| **Number of samples** | 1334 | 1196 | 1334 | 1196 |

Note:^*^ *p < 0.05,*^**^ *p < 0.01,*^***^ *p < 0.001;*The values in parentheses are t-values.

6.3 Comparison of the impact of epidemics on trade between industrial and service countries

Similarly, we use the ratios of industrial added value to GDP and service added value to GDP published by the World Bank in 2018 as the standards to measure industrial and service countries and define those countries with a ratio greater than 25% as the industrial and service group respectively, and then make estimation and comparative analysis in each group. The estimated results are shown in Table 8.

As can be seen from the estimated results in Table 7, the epidemic has a significant negative impact on trade growth and trade ratio in both the industrial group and the service group. The impact of epidemic duration was consistent with the original estimates. By further observing the absolute value of the regression coefficient, we can see that the impact on the trade of industrial countries is slightly less than that on the trade of service countries, but industrial countries are more vulnerable to the long-term impact of the epidemic to form a long-term trade diversion effect, or it is more difficult for industrial countries to recover their trade after the epidemic is over than that of service countries. In recent years, the proportion of service trade in global trade has been increasing. Service countries with high-tech, knowledge products and emerging service industries as the main players have lower substitutability of their dominant industries. Even if there is a more serious short-term trade contraction, there will be no significant industrial outflow phenomenon, and the corresponding trade share can still be recovered after the end of the epidemic. There will be no long-term trade diversion effect; However, countries that mainly export industrial products are often less developed countries, and the growth of service trade is slow. Even if they can survive a certain trade contraction in the short term due to inventory protection, they are more likely to form industrial outflow in the long term trade contraction, and thus form long-term trade diversion and lose market share. This also shows that when the epidemic occurs, we should pay special attention to the outflow of industrial production, especially the middle and low-end manufacturing industry, increase investment, enhance the technological growth in industrial production, improve the added value of Chinese industrial production in the international value chain, reduce the substitutability of export products, and ensure the stable development of industrial structure.

Non-epidemic diseases have a positive effect on the trade ratio of industrial countries and a negative effect on the trade ratio of service countries. The effect of the duration of non-pandemic diseases on the trade ratios of industrial and service countries is also reversed. This suggests that non-pandemic diseases, such as common influenza, do not halt industrial activity, but may shrink service activity.

In conclusion, both epidemic and non-epidemic diseases have a greater impact on service countries in the short term. In the medium to long term, the impact of epidemic diseases on industrial countries is greater, while the impact of non-epidemic diseases on service countries is still greater.

**Table 8.** Group estimation results: industrial countries and service countries.

|  | **Model II Industrial countries** | **Model II Service country** | **Model Ⅳ Industrial countries** | **Model Ⅳ Service countries** |
| --- | --- | --- | --- | --- |
|  | *TradeGrowth* | *TradeGrowth* | *TradeRatio* | *TradeRatio* |
| ***EDI*** | -0.0000130^***^ | -0.0000152^**^ | -0.000000558^***^ | -0.000000371^***^ |
|  | (-3.51) | (-3.17) | (-4.54) | (-4.34) |
| ***NEDI*** | 0.00477 | 0.00673 | 0.000299^**^ | -0.000505^***^ |
|  | (1.38) | (1.21) | (2.60) | (-5.12) |
| ***EDITime*** | 0.0309^***^ | 0.00917 | 0.00190^***^ | 0.000762^**^ |
|  | (3.87) | (0.68) | (7.18) | (3.16) |
| ***EDITime^2^*** | -0.00334^***^ | 0.000390 | -0.000115^***^ | -0.000107^*^ |
|  | (-3.96) | (0.16) | (-4.10) | (-2.41) |
| ***NEDITime*** | 0.00239 | -0.0630 | -0.00325^***^ | 0.00305^***^ |
|  | (0.11) | (-1.65) | (-4.44) | (4.48) |
| ***NEDITime^2^*** | 0.00600 | 0.0118 | 0.000457^**^ | -0.00141^***^ |
|  | (1.37) | (0.86) | (3.13) | (-5.81) |
|  |  |  |  |  |
| **Term of constant** | 0.908^***^ | 1.038^***^ | 0.0111^***^ | 0.00259^***^ |
|  | (22.13) | (26.98) | (8.17) | (3.78) |
| **Number of samples** | 1311 | 1219 | 1311 | 1219 |

Note:^*^ *p < 0.05,*^**^ *p < 0.01,*^***^ *p < 0.001;*The values in parentheses are t-values.

6.4 Summary

To sum up, the original baseline estimation results are relatively robust. In addition, through the analysis of the new grouping structure, the following conclusions are further drawn: the impact of epidemics on the trade of large countries is greater than that of small countries, and the impact of epidemics on the trade of large countries is more likely to form a long-term trade diversion. The impact of epidemic disease on the trade of agricultural countries is less than that of non-agricultural countries, and non-agricultural countries are more likely to form a long-term trade diversion while suffering the impact of epidemic disease. In the short term, the trade impact of the epidemic on industrial countries is less than that on service countries. In the medium and long term, industrial countries are more likely to have a long-term trade diversion than service countries. Therefore, for the country such as China which is both a large economy and industrial country, it is much more important to adopt advancing policies to ensure the stability, transformation and upgrading of industrial structure in the long run of the epidemic, avoiding the trade diversion to harm the industry structure too much.

7. Conclusions

This paper comprehensively reviews the theoretical mechanism of the impact of epidemic disease on trade and the formation of long-term trade diversion effect, and obtains strongly robust estimation results, which can provide supplementary research literature and general empirical evidence for whether epidemic disease will impact a country's trade and lead to trade shock or trade diversion. According to the conclusion of this study, the impact of epidemic disease has a deep impact on the industrial structure of a country, which would affect the stability of a country's long-term economic growth. The outbreak of COVID-19 in 2019 and recurrence in the autumn and winter of 2020 prompted all countries around the world to strengthen epidemic prevention and control efforts, while some countries were excepting China have yet to recover their pre-epidemic economic and trade growth in the post-epidemic era. In April 2021, the International Monetary Fund proposed its World Economic Outlook that many countries will not recover their pre-pandemic GDP growth levels until 2023, indicating that the general impact of COVID-19 on countries' economic and industrial structures may have a long-lasting impact. China's experience of achieving positive economic and trade growth in a short period of time during 2021 provides a blueprint for other countries' foreign trade recovery. Even though, we can still find that the continued impact of the epidemic on the economy and trade would not so easy to erased, which urge all countries to strengthen international communication and deepen international cooperation. This includes: First, economic diplomacy should be expanded and effective implemented, advocating free trade and multilateral trade, opposing trade protectionism, and gradually resuming trade exchanges. Second, we need to accelerate the building of a community with a shared future for mankind, advocate an international political and economic order featuring mutually beneficial cooperation and win-win development.

This paper shows a brief picture and simple empirical identification of that whether a endogenous economic shock could impact trade and lead to trade shock or trade diversion. With the COVID-19 turns to Omikjon, the worldwide major countries have announced relaxing prevention and control, urge the global trade to recovery. Even though, due to the experience observed in this paper, it is might be seen that lots of countries will find they have lost their trade partners and share of the worldwide trade in next several years. COVID-19 could be so different from the epidemics chosen to the estimation in this paper, because it impacts such a worldwide range and lasting really a long time. Both would make COVID-19 a historical impact (Kostova et al., 2019; Wanjala and Kevin, 2020; Brenton et al., 2020; Welfens and Paul, 2020; Megits et al., 2020; Antràs Pol et al., 2020)[17-22], and more research will find how greatly and deeply it will influence the global trade and industrial structure. This paper use historical experience is to just provide some reference to understand and foresee the huge challenges would happen to many countries in the future.

References

1. Fernandes, Ana and Tang, Heiwai, How Did the 2003 SARS Epidemic Shape Chinese Trade? May 15, 2020, Available at SSRN: https: //ssrn.com/abstract=3603699

2. Yu, Zhen, Yao Xiao, and Yuankun Li. The response of the labor force participation rate to an epidemic: Evidence from a cross-country analysis. Emerging Markets Finance and Trade, 2020, 56(10): 2390-2407.

3. Dai, Chenghao. The impact of the epidemic on the economy--an analysis from the perspective of unemployment rate and household consumption. Highlights in Business, Economics and Management, 2022, 2: 81-94.

4. Gruszczynski, Lukasz. The COVID-19 pandemic and international trade: Temporary turbulence or paradigm shift?. European Journal of Risk Regulation, 2020, 11(2): 337-342.

5. Arnon, Alexander, John Ricco, and Kent Smetters. Epidemiological and economic effects of lockdown. Brookings Papers on Economic Activity, 2020, 2020(3): 61-108.

6. Handfield, R.B., Graham, G. and Burns, L. Corona virus, tariffs, trade wars and supply chain evolutionary design", International Journal of Operations & Production Management, 2020, 40(10): 1649-1660. https: //doi.org/10.1108/IJOPM-03-2020-0171

7. Queiroz, Maciel M., et al. Impacts of epidemic outbreaks on supply chains: mapping a research agenda amid the COVID-19 pandemic through a structured literature review. Annals of operations research, 2020: 1-38.

8. White, Alexandre IR. Historical linkages: epidemic threat, economic risk, and xenophobia. The Lancet, 2020: 1250-1251.

9. Delivorias, Angelos, and Nicole Scholz. Economic impact of epidemics and pandemics. European Parliamentary Research Service, 2020.

10. Eichenbaum, Martin S., Sergio Rebelo, and Mathias Trabandt. The macroeconomics of epidemics. The Review of Financial Studies, 2021, 34(11): 5149-5187.

11. Huang Yueheng, Luo Zhengjing, Feng Zongxian. On endogenous technical barriers to trade in commodity quarantine. Journal of Quantitative & Technical Economics, 2004, (05) : 25-30.

12. Leadon D. P., Herholz C. Globalisation of Trade and the Spread of Infectious Disease. Equine vet. Educ. Manual, 2009, 8.

13. Shi Changhua, Gong Hanying. Preliminary study on improving the system of technical trade measures in response to public health emergencies in China. Port Health Control, 2013, 18 (03) : 1-4.

14. Huang Zhaoyin. SARS challenges China's foreign trade. Journal of Wuhan University (Social Science Edition), 2003, (04) : 408-409.

15. Shao Bai, Wei Shan, Liu Mingjie. Study on the impact and Countermeasures of International Public health Emergencies on international trade. Port Health Control, 2019, 24 (04) : 1-3.

16. Perrings C., Levin S., Daszak P. The Economics of Infectious Disease, Trade and Pandemic Risk. 2018.

17. Kostova, Deliana, et al. Long‐distance effects of epidemics: Assessing the link between the 2014 West Africa Ebola outbreak and US exports and employment. Health economics, 2019, 28(11): 1248-1261.

18. Wanjala, Kevin. The Economic Impact Assessment of the Novel Coronavirus on Tourism and Trade in Kenya: Lessons from Preceding Epidemics. Finance & Economics Review, 2020, 2(1): 1-10.

19. Brenton, Paul; Chemutai, Vicky. Trade Responses to the COVID-19 Crisis in Africa. Trade and COVID-19 Guidance Note. World Bank, Washington, DC., 2020.

20. Welfens, Paul JJ. Macroeconomic and health care aspects of the coronavirus epidemic: EU, US and global perspectives. International Economics and Economic Policy, 2020, 17(2): 295-362.

21. Megits, Nikolay, Inna Neskorodieva, and Julian Schuster. Impact assessment of the COVID-19 on trade between Eastern Europe and China. Journal of Eastern European and Central Asian Research (JEECAR), 2020, 7(3): 385-399.

22. Antràs, Pol, Stephen J. Redding, and Esteban Rossi-Hansberg. Globalization and pandemics. No. w27840. National Bureau of Economic Research, 2020.

23.Dimitri, Nicola. The Economics of Epidemic Diseases.PLOS ONE.2015(9).

24.Gong Mengchun, Liu Li,Wu Chen, Yang Yue,Shen Yang, Li Jie, etc.Conducting clinical studies during the epidemics of communicable diseases: perspectives of methodology and health economics.Journal of Southern Medical University.2020 (3):353-357.

25.Boucekkine, Raouf Carvajal, Andres Chakraborty Shankha, Goenka Aditya. The economics of epidemics and contagious diseases: An introduction. Journal Of Mathematical Economics. 2021(03).

26.Sung Janet. Fighting the oral disease epidemic: a call for more behavioral economics research. Perspectives In Public Health.2016(03).

27.Xing W B, Tian K. Research progress on economic impact of epidemics and intervention policies. Economic Review,2020,(07):113-128.

28.Yin Jing, Wang Chunchao. Theoretical and empirical research progress of epidemiological economics. Economic Review,2020,(07):99-112.

29.Xi Chen. Advances in theoretical and empirical research of epidemiological economics Global Lecture Series on Chinese Economy.2020-05-20..

30.Thomas A. Garrett, Yunlu Tong. The economics of Pandemics: The 1918 pandemic Influenza and its Modern Significance. Financial Markets Research,2020,(03):37-50.

31. Clausing K. A. Trade Creation and Trade Diversion in the Canada–United States Free Trade Agreement. Canadian Journal of Economics/Revue Canadienne D'économique, 2001, 34(3): 677-696.

32. Bohara A. K., Gawande K., Sanguinetti P. Trade Diversion and Declining Tariffs: Evidence from Mercosur. Journal of International Economics, 2004, 64(1): 65-88.

33. Guo Yan, Li Xiubin. The Increasingly Prominent Effect of Trade Diversion: A Study on the Change of the import trade pattern of Textiles and Apparel in the United States in the era of zero quota. International Trade, 2004, (07) : 37-40.

34 Zhang Bin, Zhang Shu. Trade Creation and Transfer of the United States in NAFTA: 1994~2003. World Economy, 2005, (08) : 13-23.

35. Magee C. S. P. New Measures of Trade Creation and Trade Diversion. Journal of International Economics, 2008, 75(2): 349-362.

36. Shen Hao, Yang Yong. Analysis on the trade creation and trade diversion effect of African economic integration. International Trade Issues, 2008, (04) : 49-54.

37. Datta A., Kouliavtsev M. NAFTA and the Realignment of Textile and Apparel Trade: Trade Creation or Trade Diversion?. Review of International Economics, 2009, 17(1): 172-186.

38. Sun L., Reed M. R. Impacts of Free Trade Agreements on Agricultural Trade Creation and Trade Diversion. American Journal of Agricultural Economics, 2010, 92(5): 1351-1363.

39. Baylis K., Perloff J. M. Trade Diversion from Tomato Suspension Agreements. Canadian Journal of Economics/Revue Canadienne D'économique, 2010, 43(1): 127-151.

40. Cao Liang, Jiang Hongbin, Chen Xiaohong. Research on the trade creation and diversion effect of CAFTA. Macroeconomic Research, 2013(06) : 29-34+71.

41. Yang S., Martinez-Zarzoso I. A Panel Data Analysis of Trade Creation and Trade Diversion Effects: The Case of ASEAN–China Free Trade Area. China Economic Review, 2014, 29: 138-151.

42. Dai M., Yotov Y. V., Zylkin T. On the Trade-diversion Effects of Free Trade Agreements. Economics Letters, 2014, 122(2): 321-325.

43. Cheong J., Kwak D. W., Tang K. K. Can Trade Agreements Curtail Trade Creation and Prevent Trade Diversion?. Review of International Economics, 2015, 23(2): 221-238.

44. Magee C. S. P. Trade Creation, Trade Diversion, and the General Equilibrium Effects of Regional Trade Agreements: A Study of the European Community–Turkey Customs Union. Review of World Economics, 2016, 152(2): 383-399.

45. Xu Fen, Liu Hongman. Study on the trade creation and trade diversion Effect of China's agricultural imports in Free Trade Zone: Based on SYSGMM Estimation of import demand model. Issues of Agricultural Economy, 2017, 38 (09) : 76-84+111-112

46. Brenton P. Anti-dumping Policies in the EU and Trade Diversion. European Journal of Political Economy, 2001, 17(3): 593-607.

47. Chen Hanlin. Analysis and countermeasures of the trade transfer effect of US anti-dumping against China. International Trade, 2008, (09) : 18-22.

48. Xiang Hongjin. Study on the trade restriction Effect and trade diversion Effect of Foreign Anti-dumping Measures against China. Journal of Quantitative & Technical Economics, 2008, 25 (10) : 75-86.

49. Liu Graviti, Shao Min. The Trade Diversion Effect of Indian Anti-dumping against China: An Empirical Analysis Based on Product Perspective. International Economic and Trade Exploration, 2009, 25 (09) : 48-53.

50. Park S. The Trade Depressing and Trade Diversion Effects of Antidumping Actions: The Case of China. China Economic Review, 2009, 20(3): 542-548.

51. Sun Ming, Yang Shihui. Trade diversion Effects of Countervailing Claims: An empirical analysis based on panel Data. Finance and Trade Research, 2011, 22 (05) : 65-70.

52. Liu G Yi, Cao Jie. The Trade Diversion Effect of EU Anti-dumping against China: An Empirical Analysis Based on Product Perspective. International Trade Review, 2011, (07) : 91-101.

53. Zhang Yong. Analysis of the trade diversion effect of the U.S. anti-dumping Complaint. International Economic and Trade Exploration, 2013, 29 (04) : 16-26

54. Hao Liang. Manufacturer decision order, trade diversion effect and determination of optimal anti-dumping duty. Issues of International Trade, 2017, (05) : 139-152.

55. Headey D. Rethinking the Global Food Crisis: The Role of Trade Shocks. Food Policy, 2011, 36(2): 136-146.

56. Liu Hongguang, Zhang Jie, Zhu Zhongxiang, Zhang Zimeng. Quantitative analysis of global industrial trade transfer before and after financial crisis. Economic Geography, 2019, 39 (01) : 96-103. (in Chinese)

57. Kim, Jaewook, et al. Effects of epidemic disease outbreaks on financial performance of restaurants: Event study method approach. Journal of Hospitality and Tourism Management, 2020, 43: 32-41.

58. Loayza, Norman. Costs and trade-offs in the fight against the COVID-19 pandemic: A developing country perspective. World Bank Research and Policy Briefs, No.148535, 2020.

59. Aum, Sangmin, Sang Yoon Tim Lee, and Yongseok Shin. Inequality of fear and self-quarantine: Is there a trade-off between GDP and public health?. Journal of Public Economics, 2021, 194: 104354. https: //doi.org/10.1016/j.jpubeco.2020.104354

60. Bontempi, Elza, et al. Can commercial trade represent the main indicator of the COVID-19 diffusion due to human-to-human interactions? A comparative analysis between Italy, France, and Spain. Environmental research, 2021, 201: 111529.

61. Anderson T. W., Cheng H.. Formulation and Estimation of Dynamic Models Using Panel Data. Journal of Econometrics, 1982, 18(1): 47-82.

62. Bai J. Panel Data Models With Interactive Fixed Effects. Econometrica, 2009, 77(4): 1229-1279.
