# Supplementary material for: Trade shocks and trade diversion due to epidemic diseases: Evidence from 110 countries
Source: PLoS One. 2024 May 31;19(5):e0301828. doi: 10.1371/journal.pone.0301828 (PMC11142499; doi:10.1371/journal.pone.0301828)
Supplement: Attachment — Submitted filename: Response to Reviewers.docx [file pone.0301828.s007.docx]

**Cover Letter**

Dear Editor: Thank you very much for your revision suggestions!

Three issues raised by the editor about our manuscript have been corrected:

1) All within-text citations need to be reformatted throughout the manuscript. Citations are reoccurring in two formats, for example (Fernandes et al., 2020) [1] and (Yu Zhen et al., 2020; Dai Chenghao, 2022) [2–3]. I would suggest the authors keep the numbered citations ([1], [2-3]) that follow the guidelines of PLOS ONE and remove the citations with name and year formatting (Yu Zhen et al., 2020; Dai Chenghao, 2022).

To solve this problem, we have unified the format according to the PLOS ONE specification.

2) Please check all the references and ensure they match the citations throughout the manuscript. For example, check reference no. 86.

We made one-to-one corrections to the references.

3) The numbering of headings and sub-headings needs to be updated. For example, after sub-heading 4.3.3 is 4.4.4.

We verified and corrected numbering of headings and sub-headings.

Liu Bangfan

2024-03-02
