# Supplementary material for: Trade shocks and trade diversion due to epidemic diseases: Evidence from 110 countries
Source: PLoS One. 2024 May 31;19(5):e0301828. doi: 10.1371/journal.pone.0301828 (PMC11142499; doi:10.1371/journal.pone.0301828)
Supplement: Attachment — Submitted filename: Referee report-PLOS ONE.pdf [file pone.0301828.s001.pdf]

Epidemic Disease Caused Trade Shocks and Long-term Trade Diversion: Evidence from 110 Countries in 1996—2018

Referee report

1. Theme of the paper

Title: ***Epidemic Disease Caused Trade Shocks and Long-term Trade Diversion: Evidence from 110 Countries in 1996—2018***

Suitable/not suitable/ Need Improvements: *Need Improvements*

- *Scope of this topic is very broad, need to be more specific*
- *Better to highlight methodologies that were used for your predictions*

2. Abstract

- *Need to rearrange your abstract as follows: Purpose, Methodology and Design , Findings, Practical implications and Originality*
- *Purpose of your study not clear, need to explain clearly*
- *Model validation part is missing, need to discuss it under your abstract also*

3. Introduction/Background information

Considering the following facts this section needs development:

- *Background of the study is not enough. Need to focus more with your objectives.*
- *Need to explain following points:*
    - *What is the effect of the current pandemic in the international trade?*
    - *What do you think have been the main consequences of pandemics?*
- *Clearly define the research problem*
- *State your research aims, objectives and questions*
- *Explain the significance of your study*
- *Identify the limitations of your research*
- *"Summary of literature" part is not enough, need to discuss more based on your literature findings and identified limitations*

4. Objectives

- *Objectives are very general, need to define objectives and specified objectives clearly*
- *Novelty of the study not clear.*

5. Study area and Data

- *What are the main reasons to select this study area?*
- *As we know, the epidemic disease caused significant global trade shocks and long-term trade diversion during covid pandemic from 2020-2022. So, better to consider your study period up to 2022.*
- *Data proposing part is not clear, what are the reasons to use yearly data*

6. Result and discussion

- *Need to do behavioral analysis to identify the behaviors of the data patterns*

7. Conclusions and recommendations

- *Need to explain your findings and novelty of the study more briefly.*
- *Need to state the significance of results.*
- *Discuss your study findings clearly.*

8. References

- *Study depend with only few references, so need to add more recent references with different methodologies*

9. **Overall Judgment**:

Acceptable for publication with major revisions
